# Engineering Prodrug Nanomedicine for Cancer Immunotherapy

**DOI:** 10.1002/advs.202002365

**Published:** 2020-10-25

**Authors:** Bin Yang, Jing Gao, Qing Pei, Huixiong Xu, Haijun Yu

**Affiliations:** ^1^ State Key Laboratory of Drug Research & Center of Pharmaceutics Shanghai Institute of Materia Medica Chinese Academy of Sciences Shanghai 201203 China; ^2^ Department of Medical Ultrasound Shanghai Tenth People's Hospital Ultrasound Research and Education Institute Tongji University School of Medicine Tongji University Cancer Center Shanghai 200072 China

**Keywords:** cancer immunotherapy, combination immunotherapy, nanomedicine, prodrugs, stimuli‐responsive

## Abstract

Immunotherapy has shifted the clinical paradigm of cancer management. However, despite promising initial progress, immunotherapeutic approaches to cancer still suffer from relatively low response rates and the possibility of severe side effects, likely due to the low inherent immunogenicity of tumor cells, the immunosuppressive tumor microenvironment, and significant inter‐ and intratumoral heterogeneity. Recently, nanoformulations of prodrugs have been explored as a means to enhance cancer immunotherapy by simultaneously eliciting antitumor immune responses and reversing local immunosuppression. Prodrug nanomedicines, which integrate engineering advances in chemistry, oncoimmunology, and material science, are rationally designed through chemically modifying small molecule drugs, peptides, or antibodies to yield increased bioavailability and spatiotemporal control of drug release and activation at the target sites. Such strategies can help reduce adverse effects and enable codelivery of multiple immune modulators to yield synergistic cancer immunotherapy. In this review article, recent advances and translational challenges facing prodrug nanomedicines for cancer immunotherapy are overviewed. Last, key considerations are outlined for future efforts to advance prodrug nanomedicines aimed to improve antitumor immune responses and combat immune tolerogenic microenvironments.

## Graphical abstract

Prodrug‐based nanomedicine is exploited for boosting cancer immunotherapy by simultaneously eliciting the antitumor immunogenicity and reversing the immunosuppressive tumor microenvironment. In this review article, the cutting‐edge advances, challenges, and perspectives of prodrug nanomedicine are overviewed. More efforts should be devoted to exploiting prodrug nanomedicine for enhancing immune response and combating immune tolerance in the future.

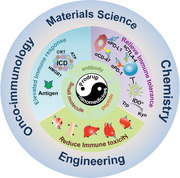

## 1 Introduction

Clinical strategies in cancer immunotherapy typically focus on either modulating the tumor immune microenvironment or inducing an antitumor immune response. Together, these approaches have made some impressive advances in the clinical management of cancer in recent years. In some cases, cancer immunotherapy can induce an antigen‐specific immune response to cause tumor regression and elicit the immune memory effects for tumor metastasis and reoccurrence suppression.^[^ ^1^, ^2^ ^]^ However, this effect can be difficult to achieve with current cancer immunotherapy approaches, which are hindered by the inherently low immunogenicity of tumor cells:^[^ ^3^, ^4^ ^]^ i) the tumor microenvironment secretes cytokines to inhibit the maturation of dendritic cells (DCs), resulting in an attenuated presentation of tumor‐associated antigens (TAAs);^[^ ^5^ ^]^ and ii) tumor cells reduce immune responses by downregulating the expression of major histocompatibility complex I (MHC‐I), which can decrease the efficacy with which antigen presenting cells (APCs) present TAA to the T lymphocytes.^[^ ^6^ ^]^ Thus, extensive efforts had been aimed to improve antitumor immunogenicity by promoting antigen presentation, activating T cells, and inducing immunogenic cell death (ICD) of the tumor cells.^[^ ^6^, ^7^ ^]^ Agonists of stimulator of the interferon gene (STING),^[^ ^8^ ^]^ and Toll‐like receptor (TLR)^[^ ^9^ ^]^ have been also exploited for improving cancer immunotherapy. These strategies act by facilitating type I interferon (e.g., IFN‐*α* and IFN‐*β*) secretion.

However, clinical responses to therapeutic strategies aimed to enhance antitumor immunogenicity have been largely unimpressive, for several potential reasons: i) Tumors suppress the function of the cytotoxic T lymphocytes (CTLs) via immune checkpoints whose normal function is to maintain immune balance and avoid autoimmune disease, including programmed death protein‐1 (PD‐1), programmed death ligand 1 (PD‐L1), cytotoxic T lymphocyte‐associated protein 4 (CTLA‐4), lymphocyte activation gene 3 (LAG‐3), etc.^[^ ^10^ ^]^ ii) Tumors secrete various cytokines and negative regulators (e.g., indoleamine 2,3‐dioxygenase‐1 (IDO‐1), cyclooxygenase‐2 (COX‐2), transforming growth factor‐*β* (TGF‐*β*), etc.) to induce an immunosuppressive tumor microenvironment and^[^ ^11^, ^12^ ^]^ a local abundance of immunosuppressive cells, such as myeloid‐derived suppressor cells,^[^ ^13^ ^]^ and intratumoral T‐regulatory cells.^[^ ^14^ ^]^ iii) Tumors can downregulate the impact of inflammatory immune signals through various metabolic pathways.^[^ ^15^ ^]^ For example, the hypoxic environment of tumors accelerates the production of lactic acid to produce an acidic microenvironment, thereby promoting polarization of tumor‐associated macrophages (TAM), resulting in transformation of the TAM into M2 phenotype for promoting tumor growth.^[^ ^16^, ^17^ ^]^

Efforts to reverse the immunosuppressive tumor microenvironment (ITM) are another crucial strategy for improving cancer immunotherapy. For instance, immune checkpoint blockade (ICB) therapy significantly elongates the survival of some treated cancer patients.^[^ ^18^ ^]^ Immune checkpoint inhibitors restore the function of CTLs by blocking the immune checkpoints, allowing them to kill the tumor cells.^[^ ^19^ ^]^ To date, the Food and Drug Administration (FDA) of USA has approved 5 antibody‐based immune checkpoint inhibitors, including pembrolizumab targeting PD‐1 and atezolizumab targeting PD‐L1. Along with antibody‐based immune checkpoint inhibition, small molecule immune modulators also play an important role in modulating the ITM.^[^ ^20^ ^]^ For instance, inhibitors of IDO‐1^[^ ^11^ ^]^ have been extensively exploited as a strategy to improve cancer immunotherapy.^[^ ^21^ ^]^

Despite the great potential advantages of immunotherapy for clinical cancer treatment, such therapeutics carry an inherent risk of off‐target effects in the rest of the body. For instance, “immune cytokine storm” is sometimes caused by ICB therapy due to off‐target binding of immune checkpoint inhibitors, whose receptors are also expressed on the surface of the vascular endothelium, mesenchymal stem cells, epithelium, and muscle cells.^[^ ^22^ ^]^ Furthermore, many small molecule drugs are quickly metabolized following systemic administration, leading to difficulty achieving therapeutic concentrations at the site of the tumor.^[^ ^23^ ^]^ In addition, cancer vaccines alone are typically insufficient to promote immune response, and require the help of immune adjuvants. Thus, properly designing advanced drug delivery systems may be necessary to deliver highly efficient cancer immunotherapy. Several recent studies have suggesting that targeting intracellular organelles, such as the endoplasmic reticulum, can dramatically improve the effectiveness of cancer immunotherapy.^[^ ^24^ ^]^ Hence, a multifunctional delivery system that protects encapsulated drugs and improves targeting to tumor tissues may enable more efficient delivery of oncotherapeutics.

**Scheme 1:**
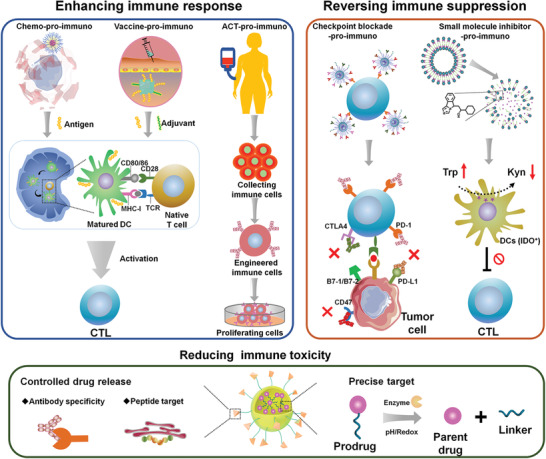
Schematic illustration of various kinds of prodrug nanomedicine for promoting cancer immunotherapy and their roles in immunotherapy. The prodrug‐based nanoplatforms can be engineered to boost antitumor immunogenicity, reverse the immunosuppressive tumor microenvironment, or reduce the immune side effects of immunotherapeutics.

In the context of the recent expansive development of nanotechnologies, the integration of nanomedicine in the field cancer immunotherapy has become a topic of widespread interest. Nanomedicines can enhance therapy efficacy and minimize immune‐related side effects.^[^ ^2^, ^25^ ^]^ The tumor‐associated vasculature consists of relatively discontinuous endothelial cells that do not have a smooth muscle layer, ultimately leading to high permeation.^[^ ^26^ ^]^ Nanoparticles (NPs) can thus passively target and be retained in tumor tissue, a phenomenon called the enhanced permeability and retention (EPR) effect of solid tumors.^[^ ^27^, ^28^, ^29^ ^]^ In addition, nanoparticles can greatly enhance the stability of encapsulated cargo.^[^ ^30^, ^31^, ^32^, ^33^ ^]^ However, conventional NPs have several shortcomings, including risks of immunogenicity, accidental drug leakage during circulation, and low drug loading ability.^[^ ^34^ ^]^ To address above challenges, prodrug nanoplatforms integrating the advantages of prodrugs and nanotechnology have recently been exploited as a strategy to improve upon conventional immunotherapy of cancer.^[^ ^34^, ^35^ ^]^ In comparison with nanoparticle‐mediated, noncovalent encapsulation of free drugs, stimulus‐responsive prodrug nanoparticles have a pharmaceutic advantage. Such particles can be tuned to minimize accidental drug leakage and to control drug release profiles through the use of chemical linkers.^[^ ^36^, ^37^ ^]^ For example, serval small molecule chemotherapeutic drugs modified with oligolactides or unsaturated fatty acid to fabricate prodrugs can be simply encapsulated or directly self‐assembled into nanoparticles.^[^ ^38^, ^39^ ^]^ The synthetic prodrugs exhibit an excellent compatibility with nanoplatforms.^[^ ^40^ ^]^ Compared with solution‐based free drugs, prodrug nanomedicines via mediating the pharmacokinetic profiles of chemotherapeutic drugs showed more effective therapeutic efficacy as well as enhancement of tolerability, which significantly enhanced the therapeutic index for drugs.^[^ ^41^ ^]^ Furthermore, prodrug nanoplatforms can integrate multiple immune modulators with variable chemophysical properties and pharmacokinetic profiles into a single nanoplatform to provide synergistic immunotherapy.^[^ ^3^, ^42^ ^]^ In this review article, we summarize the emerging applications of prodrug nanomedicines as a strategy to improve cancer immunotherapy. We also discuss challenges and perspectives related to the development of nanomedicine‐based prodrug approaches to cancer immunotherapy (**Scheme** **1**).

## 2 Prodrug Nanomedicine for Improved Cancer Immunotherapy

Systemic delivery of immune modulators into solid tumors and tumor cells is hindered by a series of pathophysiological barriers including rapid blood clearance, inadequate accumulation at the tumor site, and toxicities for healthy tissues. Together, these difficulties can contribute to disappointing efficacy of immunotherapies. Multifunctional nanoplatforms are rationally designed to help overcome these physiological barriers to enable tumor‐specific drug delivery and improve bioavailability by prolonging blood circulation time, improving tumor accumulation (via the EPR effect), and mitigate the adverse effects due to nonspecific accumulation in healthy tissues.

Recent evidence suggests that prodrug‐based nanomedicines with high drug encapsulation efficiencies can reduce excipient‐associated side effects.^[^ ^43^, ^44^ ^]^ Prodrugs are chemically designed to be activated in a spatial‐, temporal‐, or dosage‐controlled fashion, allowing tunable pharmacokinetic profiles and reduced nonspecific toxicity in normal tissues. Importantly, prodrugs can be designed to, when activated, induce various elements of the immune system to enhance the effects of immunotherapy. Prodrug nanomedicines thus represent an attractive strategy to the efficient administration of immunotherapeutics for the treatment of cancer. They combine several key advantages of nanotechnology, including long circulation time, good bioavailability, drug codelivery with elements of prodrugs, such as controlled drug retention and release. Thus, prodrug nanomedicines represent a promising drug delivery approach to immunotherapy (**Table** **1**).

**Table 1:** An overview of prodrug nanomedicine for cancer immunotherapy

Main type	Active component in prodrug	Delivery platform	Responsive condition	Assisted ingredient	Immunological effects	Refs.
Chemotherapy	Dox	HA‐based NPs	MMP‐2	*α*‐PD1	Enhance ICD‐associated immunogenicity and reverse immunosuppression	^[^ ^57^ ^]^
	OXA	Core–shell magnetic NPs	GSH	–	Enhance ICD‐associated immunogenicity and downregulate the PD‐L2 expression	^[^ ^59^ ^]^
	PTX	PTX prodrug nanoparticle	Esterase		Protecting the activation of immune cells and regulating immune response	^[^ ^64^ ^]^
	CTX	Prodrug‐formulated liposome	Esterase		Enhancing M1‐type macrophage and reprogram the immunosuppressive TME	^[^ ^38^ ^]^
	OXA+DHA	Polymer core–shell nanoplatform	GSH	*α*‐PDL1	Enhance ICD‐associated immunogenicity and reverse immunosuppression	^[^ ^69^ ^]^
Vaccine	Neoantigens, CpG oligonucleotide	Vaccine nanodiscs composed of synthetic high‐density lipoprotein	Redox	*α*‐PD1	Enhance the DC maturation by controllable antigen presentation and deregulation of immunosuppression	^[^ ^74^ ^]^
	Neoantigen, adjuvant	Redox‐responsive polycondensate neoepitope	GSH		Specific enhanced T cell response	^[^ ^77^ ^]^
	TLR7/8 agonist	Polymeric nanogels	Amide bond	Antigen	Localized immune activation	^[^ ^84^ ^]^
	TLR7/8 agonist (IMDQ)	Amphiphilic block copolymer micelle	Acidic		Improve the lymphatic drainage and activate DCs	^[^ ^86^ ^]^
ACT	*α*CD45, Interleukin‐15	PEG‐*b*‐PLL modified protein nanogels	GSH	–	Target T cell surface, reprogram the surface reduction potential of T cell and trigger the release of cytokine	^[^ ^94^ ^]^
	*α*CD205	Magnetic NCs		Antigen	Facilitate the antigen uptake and presentation	^[^ ^93^ ^]^
	Fc/mAb	pHLIP‐Fc or antibody platform	Acidic	–	Activate NK cells at tumor site	^[^ ^99^ ^]^
Small molecule Inhibitor	NLG919	PEG2k‐Fmoc‐NLG	Esterase	PTX	Reverse IDO‐mediated immunosuppression	^[^ ^103^ ^]^
	NLG919	Peptide‐drug nanoprodrug	Esterase	*α*PD‐L1	Reverse immunosuppression by PD‐L1/PD‐1 signaling pathway and IDO	^[^ ^105^ ^]^
Antibody	*α*PD‐L1	Lipid nanoparticle	Esterase	Dinaciclib, radiation therapy agent	Target and deplete the TAMCs and attenuate immunosuppression	^[^ ^112^ ^]^
	Antibody fragment	PEG‐PLGA nanoparticles	Esterase	TGF*β*1 inhibitor, TLR7/8 agonist	Restore effector T‐cell function by inhibiting the TGF*β*1 activity, recruit lymphocytes to noninflamed tumors	^[^ ^114^ ^]^
	*α*PD‐1	Magnetic nanoclusters	pH	–	Reverse immunosuppress of tumor	^[^ ^120^ ^]^
	*α*PD‐1	Dual pH‐sensitive PDPA nanoparticles	pH	CUR (NF‐*κ*B inhibitor)	Combination of PD‐1 blockade and NF‐*κ*B inhibition	^[^ ^122^ ^]^
	*α*PD‐1, *α*CTLA‐4	*α*TfR or angiopep‐2 peptide modified biopolymer scaffold	ROS	*α*TfR or angiopep‐2 peptide	Conquer BBB, improve CTLs and reduce Tregs	^[^ ^125^ ^]^
	aSIRP*α*, aCD47	Exosome derived from M1 macrophage	pH	–	Overcome immune tolerance, enhance phagocytosis	^[^ ^130^ ^]^
Binary cooperative prodrug nanomedicines	OXA Pyrolipid	Core–shell nanoscale synergistic vesicles	GSH	PPa, *α*PD‐L1	Enhance ICD‐associated immunogenicity and reverse immunosuppression	^[^ ^133^ ^]^
	OXA, PPa	Prodrug vesicles	MMP‐2, pH, GSH	*α*CD47	Enhance ICD‐associated immunogenicity and alleviate the tumor microenvironment immunosuppression	^[^ ^137^ ^]^
	Peptide for antagonist of PD‐1	Amphiphilic therapeutic peptide‐based NPs	Acidic, MMP‐2	NLG919	Reverse immunosuppression by PD‐L1/PD‐1 signaling pathway and IDO	^[^ ^143^ ^]^

### 2.1 Chemotherapeutic‐Based Prodrug Nanomedicines for Cancer Immunotherapy

Chemotherapy is the historically dominant approach for the treatment of advanced or metastatic tumors.^[^ ^45^ ^]^ In recent years, several traditional chemotherapeutic drugs (doxorubicin (DOX), oxaliplatin (OXA), paclitaxel (PTX)), which induce ICD, have shown significant potential in preventing tumor regression, metastasis, and recurrence.^[^ ^46^, ^47^ ^]^ The tumor cells undergoing ICD release immune‐stimulatory damage‐associated molecular patterns (DAMPs),^[^ ^48^ ^]^ such as calreticulin (CRT), which is released from the endoplasmic reticulum to the surface of cell membrane,^[^ ^49^ ^]^ adenosine triphosphate (ATP),^[^ ^50^ ^]^ and high mobility group box 1 (HMGB1).^[^ ^51^ ^]^ Local APCs recognize these DAMPs and subsequently become activated (presenting molecules such as CD80/86), at the same time as they encounter tumor‐associated antigens (TAA), which are presented on major histocompatibility complex (MHC). Subsequently, the activated APCs present TAA to the T lymphocytes, eliciting a protective T cell immune response.^[^ ^52^ ^]^

However, there remain some intrinsic disadvantages of chemotherapy‐based immunotherapy of cancer, such as rapid drug clearance, poor tumor targeting ability, and severe adverse effects.^[^ ^34^, ^53^ ^]^ Recent approaches in the fields of biomaterials and nanotechnology, such as prodrug‐based delivery systems, have aimed to address these drawbacks^[^ ^34^, ^54^ ^]^ to combine chemotherapy with immunotherapy for cancer treatment.

#### 2.1.1 Polymeric Prodrug‐Based Nanomedicines for Cancer Immunotherapy

**Figure 1:**
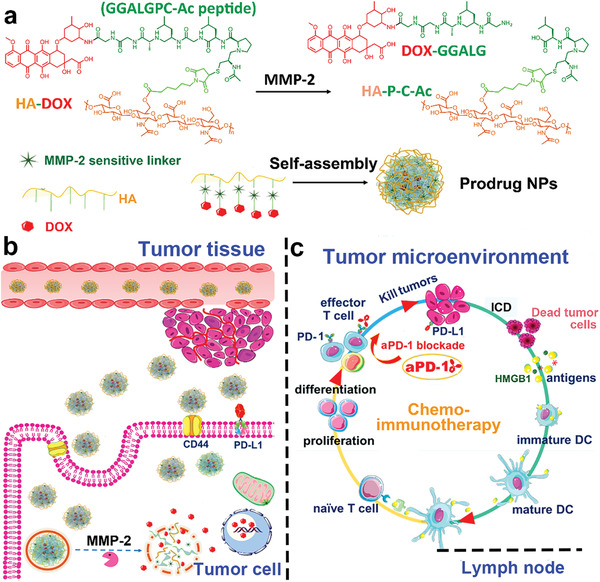
a) Chemical structure and synthesis of HA‐linker‐DOX and preparation of prodrug NPs. b) Schematic illustration of utilizing prodrug NPs to elevate immune response. c) Mechanism of chemoimmunotherapy receiving HA‐Psi‐DOX combined with *α*PD‐1. Reproduced with permission.^[^ ^58^ ^]^ Copyright 2019, American Chemical Society.

DOX, an anthracycline chemotherapeutic used for the first‐line treatment of late stage or metastatic tumors, has been well‐described as inducing ICD of tumor cells.^[^ ^55^ ^]^ T The tumors undergoing ICD are transformed from a “cold” to “hot” tumor due to improved immunogenicity.^[^ ^56^ ^]^ However, free DOX also causes severe cardiotoxicity and undesirable immune responses in normal organs. To address this general adverse effect, researchers have conjugated chemotherapeutic drugs onto polymeric nanocarriers, creating a polymeric prodrug nanomedicine to provide control over drug release kinetics (ideally reducing activity of drug in off‐target organs such as the heart). For example, Gao et al. developed a matrix metalloproteinase (MMP‐2)‐sensitive prodrug nanoplatform combined with the administration of mAb against PD‐1 (*α*PD‐1) to promote a chemotherapeutic effect (**Figure** **1**).^[^ ^57^ ^]^ The prodrugs were fabricated with a MMP‐2‐liable peptide spacer to modify the anionic polymer of hyaluronic acid with DOX. Because of the overexpression of MMP‐2 protein in the tumor environment, such prodrug nanoparticles preferentially release DOX at the tumor site to induce apoptosis and an antitumor immune response. Compared with HA‐Psi‐DOX alone, the addition of *α*PD‐1 was associated with even better performance in vivo due to reprograming the immune microenvironment via blocking the PD‐L1/PD‐1 pathway.

OXA is a widely used chemotherapeutic that is capable of inducing ICD. However, OXA causes dose‐dependent toxicity due to poor targeting and stability. To overcome this hurdle, platinum (II) can be transformed into a platinum (IV) prodrug by chemical modification with an amphiphilic polymer. Platinum (IV) prodrugs can be selectively triggered to release platinum (II) in the presence of highly reducing conditions at the tumor site. Chen et al. developed core–shell magnetic prodrug nanoparticles (ETP‐PtFeNP) that contained polymeric shells of a Pt prodrug modified with an *α*‐enolase‐targeting peptide and a core made of oleic acid‐Fe_3_O_4_ nanoparticles.^[^ ^59^ ^]^ They demonstrated that the *α*‐enolase targeting peptide‐modified shell led to impressive tumor accumulation and subsequent endocytosis, and the platinum (IV) prodrugs were cleaved to specifically release (II) in the highly reductive conditions inside tumor cells. In addition, the core of the Fe_3_O_4_ nanoparticles can not only be used for high‐sensitivity magnetic resonance imaging, but also can be activated to release ferric ions to generate cytotoxic reactive oxygen species (ROS) in the acidic tumor microenvironment.^[^ ^60^ ^]^ Subsequently, the released oxaliplatin (II) and ferric ions synergistically induce strong ICD‐associated immunogenicity via both endoplasmic reticulum (ER) and non‐ER associated pathways. Furthermore, the authors revealed that the ETP‐Pt FeNP also downregulates the expression of PD‐L2, leading to a reduction in PD‐L2‐mediated immunosuppression and significant tumor regression.^[^ ^61^ ^]^

PTX, another widely used chemotherapeutics was also employed for prodrug nanomedicine‐based cancer immunotherapy.^[^ ^62^ ^]^ PTX can modulate the tumor immune microenvironment by promoting intratumoral secretion of interleukin‐10 (IL‐10), and reversing immune escape of the tumor cells by suppressing Tregs. Furthermore, PTX induces apoptosis of the tumor cells and elicits antigen release, which facilitate the activation of the effector T cells.^[^ ^63^ ^]^ To maximize the immunotherapeutic output while avoid the reverse side effect of PTX, Tang et al. designed PTX‐based prodrug nanoparticles for chemoimmunotherapy. The PTX‐based prodrug nanoparticles displayed lower immune toxicity and improved antitumor efficacy in comparison with that of the free PTX.^[^ ^64^ ^]^ Furthermore, Wang et al. developed cabazitaxel (CTX, the semi‐synthetic derivative of the natural taxoid)‐based prodrug by conjugating CTX with docosahexaenoic acid via an ester bond. The synthetic CTX prodrug could be integrated into the liposomal nanovesicles through self‐assemble procedure. The resultant prodrug liposome displayed sustained release of CTX and decreased systemic toxicity due to the improved pharmacokinetic profiles. Most importantly, the CTX prodrug liposome can remodel the ITM by repolarizing the TAM into M1‐type macrophages for improved antitumor efficacy.^[^ ^38^ ^]^

#### 2.1.2 Prodrug‐Encapsulated‐Based Prodrug Nanomedicines

Different from polymeric prodrug nanomedicine, the polymer materials of prodrug‐encapsulated nanomedicines interact with prodrugs in a noncovalent form, and most of them serve as the auxiliary or protective components. For example, studies have shown that artemisinin induces cell apoptosis and ICD through generating ROS after the breakage of the endoperoxide bridge that is triggered by a ferrous iron.^[^ ^65^ ^]^ However, although the level of ferrous iron in tumor cells is higher than normal cells, the antitumor potential of artemisinin has been severely limited due to the instability of the endoperoxide moiety in blood.^[^ ^66^ ^]^ Therefore, the polymer shell is needed to effectively protect the encapsulated artemisinin from degradation. Furthermore, the ITM not only impairs the function of APCs, but also limits the activation of T cells, thereby greatly reducing the tumor immune response.^[^ ^67^ ^]^ The combination of two chemotherapeutic drugs (artemisinin and oxaliplatin) can synergistically produce ROS, contributing to a significant improvement in the tumor immune response.^[^ ^68^ ^]^

**Figure 2:**
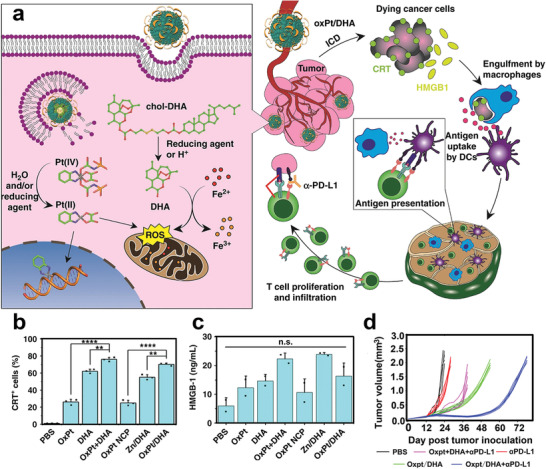
a) Left: Schematic illustration of polymer core–shell nanoplatform composed of chol‐DHA and OxPt prodrugs to release Pt(II) and DHA upon special tumor intracellular environment. Right: The mechanisms of polymer core–shell nanoplatform leading to enhanced ICD and inhibiting the activation of PD‐1/PD‐L1 pathway. Flow cytometry analysis of b) CRT cell surface expression and c) HMGB‐1 release with different treatment. d) Tumor growth curves of tumor‐bearing mice with different treatments. Reproduced with permission.^[^ ^69^ ^]^ Copyright 2019, Springer Nature.

For instance, Duan et al. developed a polymer core–shell nanoplatform to codeliver and stabilize prodrug forms of OxPt and dihydroartemisinin (DHA) (**Figure** **2**a).^[^ ^69^ ^]^ The core of this nanoparticle was an OxPt/Zn prodrug, and the lipid bilayer shell contained a conjugate of DHA with cholesterol, linked by a disulfide bond. This lipid bilayer prevented the conjugate from being degraded by water or reductants in the systemic circulation. That study found that both the OxPt/Zn prodrug and DHA prodrug could be triggered to release OxPt or DHA in the highly reducing environment of tumor. Compared to treatment with OxPt or DHA alone, the tumor cells treated with OxPt/DHA exhibited significantly greater ICD, as demonstrated by the increased ratio of CRT expression on the tumor surface and increased release of HMGB1 (Figure 2b,c).^[^ ^70^ ^]^ This core–shell nanoplatform induced long‐lasting antitumor immunity by stimulating both innate and adaptive immune response, and its antitumor efficacy was magnified by combination with *α*‐PD‐L1 (Figure 2d).^[^ ^71^ ^]^

### 2.2 Peptide‐Based Prodrug Nanoplatforms for Cancer Immunotherapy

Vaccination immunotherapy is an attractive strategy for tumor immunotherapy.^[^ ^72^ ^]^ Despite advances in cancer vaccines, some disappointing clinical trials have hindered their widespread use. These challenges may be due to unsynchronized delivery of the neoantigens and adjuvants to the tumor draining lymph nodes (dLNs), resulting in immune tolerance and decreased abundance of tumor‐specific CTLs.^[^ ^73^ ^]^ In addition, personalized tumor vaccines fabricated via neoantigens, which only target mutated tumor cells have tended to produce a short‐lived, weak anticancer immune response, potentially due to inefficient delivery platforms. To address this issue, Kuai et al. designed novel vaccine nanodiscs with synthetic high‐density lipoprotein (sHDL) loaded with conjugates of cholesterol oleate and CpG oligonucleotide (modified TLR‐9 agonist as adjuvant) and redox‐responsive neoantigens.^[^ ^74^ ^]^ The authors revealed that the vaccine nanodiscs showed excellent codelivery of modified TLR‐9 agonist and redox‐responsive neoantigens to lymphoid organs and significantly enhanced the DC maturation for controllable antigen presentation, leading to strong activation of CTLs. Most importantly, combination immunotherapy with the nanovaccine design and immune checkpoint blockade dramatically inhibited tumor growth.

**Figure 3:**
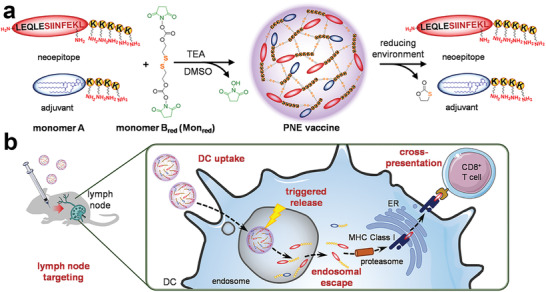
a) Preparation of the redox‐sensitive polycondensate neoepitope (PNE), and the controllable release of neoantigens and adjuvants in responsive to reducing environment. b) Mechanism of PNEs in DCs and in vivo fate. Reproduced with permission.^[^ ^77^ ^]^Copyright 2020, American Chemical Society

Another challenge for vaccination therapy is to be able to prime a strong immune response of CTLs through cross‐presentation of the antigens.^[^ ^75^ ^]^ Ideally, tumor cell antigens should escape from the lysosomal compartment of targeted cells and be loaded onto MHC‐I molecules for presentation to CD8^+^ T cells instead of being presented to CD4 ^+^ T cells after loading onto MHC‐II.^[^ ^76^ ^]^ For example, Wei et al. designed a redox‐sensitive polycondensate neoepitope (PNE) to deliver neoantigens and adjuvants for tumor vaccination immunotherapy.^[^ ^77^ ^]^ Based on a reversible polycondensation reaction, the PNEs were constructed by crosslinking neoantigen peptides (monomer A) and amphiphilic adjuvants (monomer B) with a redox‐liable linker (**Figure** **3**a). The redox‐responsive PNEs showed an effectively accumulation in draining lymph nodes and DC uptake. More importantly, the intracellular reduction of APC could trigger fast release of the neoantigens from the PNEs to facilitate endosomal escape and cross‐presentation of neoantigens, thereby greatly improving immune response (Figure 3b).

It is well‐known that the therapeutic efficiency of a cancer vaccine can be amplified by administration with adjuvants.^[^ ^78^ ^]^ So far, one widely used immune adjuvant, Toll‐like receptor agonists (TLRa), has been shown to effectively stimulate APCs to present antigen and elicit T cell immune responses. A TLR‐9 agonist based on a CpG motif oligonucleotide has been widely investigated in preclinical trials.^[^ ^79^ ^]^ However, CpG showed limited efficiency in the clinic for eliciting T cell immune responses, perhaps since TLR‐9 is expressed only in human B cells and plasmacytoid dendritic cell (pDC).^[^ ^80^ ^]^

In comparison with TLR‐9a, TLR‐7/8a displays better potential for clinical translation, since all APCs in humans express both TLR‐7 and TLR‐8.^[^ ^81^ ^]^ Nevertheless, TLRa are apt to cause systemic inflammatory toxicity when administrated in free form, due to spread into the systemic circulation.^[^ ^82^ ^]^ Although polymeric carriers have potential for improved TLR delivery by integrating both TLR agonists and antigens into one vector, noncovalent encapsulation of TLR agonists and antigens often leads to burst release of payloads after administration.^[^ ^83^ ^]^

To combat this effect, Seder et al. developed polymer‐TLR‐7/8a conjugates to explore whether the modified polymer carriers would influence the physicochemical properties of TLR‐7/8a and improve vaccine immunogenicity.^[^ ^84^ ^]^ The authors discovered that particulate polymer‐TLR‐7/8a conjugates significantly improved local retention of adjuvant in the draining lymph nodes (DLNs) and increased cellular uptake by the APCs. Compared with other formulations of TLR‐7/8a, the particulate polymer‐TLR‐7/8a reduced nonspecific distribution and improved the pharmacokinetic profile of the adjuvant.

To push forward the clinical translation of polymeric conjugates of TLR‐7/8a, Nuhn et al. designed covalently‐linked, degradable nanogels with polymerized TLR7/8 agonist for vaccination.^[^ ^85^ ^]^ They first synthesized an amphiphilic block copolymers, which could self‐assemble into nanoparticles. Then, the nanoparticles were covalently conjugated with small molecule TLR7/8 agonists via an amide bond, which offers chemically defined encapsulation. They found that the imidazoquinoline‐ligated nanogels could dramatically reduce systemic inflammation by focusing immune activation on DLNs, in contrast to soluble TLR agonists. More importantly, the localized immune activation induced by imidazoquinoline‐ligated nanogels upon coadministration of antigens remarkably enhanced the B‐ and T‐cell responses relative to free soluble TLR7/8 agonist.

**Figure 4:**
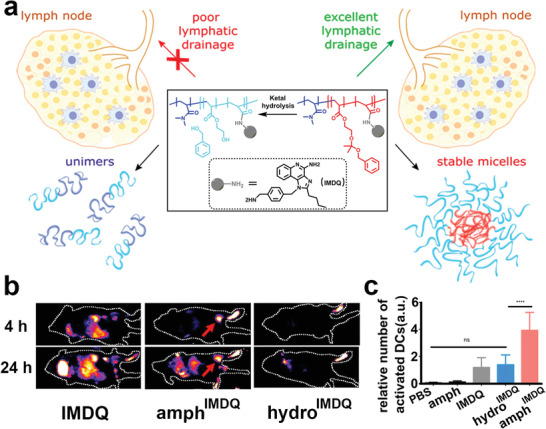
a) Scheme of the lymphatic drainage performance of TLR7/8 agonist‐conjugated micelle. b) Fluorescence imaging in vivo post footpad injection of different formulations. FACS analysis of c) activated DCs after tumor‐bearing mice receiving different treatment. Reproduced with permission.^[^ ^86^ ^]^ Copyright 2018, American Chemical Society.

To facilitate the intracellular release of TLR7/8 agonists, the same group further synthesized an acid‐activatable amphiphilic block copolymer, which was covalently conjugated with TLR7/8 agonists (IMDQ). The IMDQ‐conjugated polymer could self‐assemble into micellar nanoparticles to reduce what they describe as “wasted inflammation” during systemic circulation (**Figure** **4**).^[^ ^86^ ^]^ The micellar nanoparticles showed excellent acid sensitivity in the endosomal compartment, leading to hydrolysis into the unimer fraction by cleaved ketal bonds, nut remained stable in physiological conditions representing systemic circulation. Significantly, an amphiphilic block copolymer micelle conjugated with IMDQ (amph^IMDQ^) improved the lymphatic drainage and lymph‐node‐localized response compared to soluble IMDQ and hydro^IMDQ^ 24 h after injection, leading to a strong activation of DCs.^[^ ^87^ ^]^

### 2.3 Antibody‐Based Prodrug Nanomedicines for Adoptive Cell Therapy of Tumor

**Figure 5:**
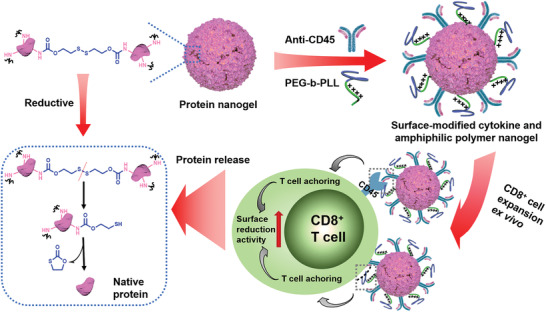
Scheme of the reductive‐responsive release of antibody from the surface of protein NGs after anchoring on the surface of T cells; fold expansion of using different modified protein NGs to stimulate the naive CD8^+^ T cells.

Adoptive cell therapy (ACT) refers to the isolation of active immune cells from a patient's body, transformation (or no modification) and expansion in vitro, and readministration into the patient for the purpose of treatment, especially against tumors.^[^ ^88^ ^]^ However, although ACT has displayed encouraging therapeutic performance against hematological tumors, it has yet to show strong immune responses against solid tumors. Fortunately, several recent studies demonstrated that the surface of the activated T cells contains more thiol groups than the inactivated T cells, and a large number of proteins or antibodies contain a thiol or disulfide bond structure.^[^ ^89^ ^]^ More importantly, the redox activity of the T cell surface can be further increased after activation with monoclonal antibodies (*α*CD3 and *α*CD28) or APCs.^[^ ^90^ ^]^ Therefore, it is a potent strategy that enables the release of immunomodulatory agents precisely by binding the redox‐sensitive nanocarrier, modified with monoclonal antibody, to the membrane of T cells. To this end, Tang et al. developed disulfide cross‐linker protein nanogels that could “backpack” protein drugs on T cells and reprogram the surface redox potential of T cells with the help of *α*CD45 and PEG‐b‐PLL (**Figure** **5**).^[^ ^91^ ^]^ Both the enhanced surface reduction potential of T cells and tumor redox microenvironment led to cleavage of the disulfide crosslinker and control drug release. The authors revealed that the *α*CD45 protein nanogels loaded with cytokines (IL‐15) expanded T cells 16‐fold, and increased the dose of cytokines 8‐fold, without apparent toxicity.^[^ ^92^ ^]^

It has been shown that antigen cross‐presentation (ACP) is a prerequisite for complete activation of T cells, and most DCs have uniquely high expression of CD205. Therefore, *α*CD205‐modified nanocarriers can be selectively recognized via DCs, thus enhancing the efficiency of MHC‐I ACP. In an elegant study, Li et al. designed a core–shell magnetic nanovaccine system (MNVs), composed of the magnetic nanoclusters (NCs) as the core, loaded with various antigens and a shell of *α*CD205‐modified cancer cell membranes that were assembled via click chemistry.^[^ ^93^ ^]^ Under the guidance of magnetic resonance imaging (MRI), the MNVs could deliver adjuvant specifically to the LNs, where they facilitated antigen uptake by the DCs. More importantly, the modified *α*CD205 on the surface of MNVs promotes the interaction between the vaccine and matured DCs, leading to superior antigen presentation and T cell activation.

In addition to CTLs, natural killer (NK) cells are an important immune regulatory cell^[^ ^94^ ^]^ that can destroy tumor cells by secreting perforin and granzyme B without a need for prior sensitization.^[^ ^95^ ^]^ Recently, the strategy of antibody‐dependent cell‐mediated cytotoxicity (ADCC)^[^ ^96^ ^]^ has attracted increased attention and even achieved successful clinical results, wherein the antibodies act as a bridge to link the NK cells with tumor cells. However, the antigens on different tumor cells show heterogeneity and significant mutation rates,^[^ ^97^ ^]^ greatly limiting the broad applicability of specific antibodies to enhance the cancer immune response. To address this dilemma, Ji et al. designed a novel ADCC (pHLIP‐Fc or antibody platform)^[^ ^98^ ^]^ by conjugating therapeutic monoclonal antibodies or Fc fragments to the N‐terminus of acid‐responsive peptides that can insert onto the surface of tumor cell membrane only in the acidic TME.^[^ ^99^ ^]^ Then, the modified cancer cells activate NK cells instead of a traditional antibody–antigen interaction. Subsequently, the activated NK cells kill the tumor cells through ADCC. The authors found that tumor growth after treatment with pHLIP‐Fc was significantly suppressed in 4T1 breast cancer and B16‐F10 melanoma tumor models in comparison with the unconjugated Fc control group.

### 2.4 Small Molecule Inhibitor‐Based Prodrug Nanomedicines for Attenuating Immune Tolerance

**Figure 6:**
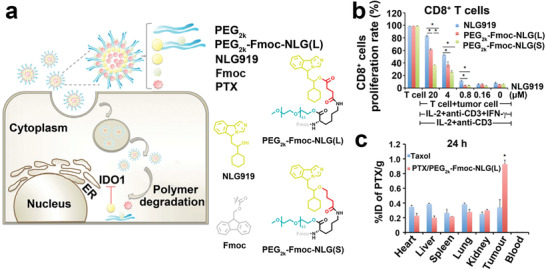
a) Schematic illustration of self‐assembled PEG_2k_‐Fmoc‐NLG919 prodrug nanomedicine. The proliferation of b) CD8^+^ T cells from Panc02 cells receiving different treatments. c) Tissue distribution in Balb/c mice treated with Taxol or PEG_2k_‐Fmoc‐NLG919 prodrug nanomedicine. Reproduced with permission.^[^ ^103^ ^]^ Copyright 2016, Springer Nature.

A strong correlation between aberrant expression of IDO‐1 and tumor progression has recently been noted.^[^ ^100^ ^]^ IDO is one of the most significant negative feedback proteins, and acts as an enzyme to transform tryptophan (Trp) to kynurenine (Kyn).^[^ ^101^ ^]^ The degradation of tryptophan suppresses the activity of the effector T cells. Meanwhile, increased accumulation of Kyn can recruit regulatory T cells (Treg) that inhibit the activity of infiltrating CTLs, leading to ITM.^[^ ^102^ ^]^ To this end, IDO inhibitor‐based nanomedicine prodrugs have recently been investigated for combating IDO‐induced immune tolerance. To illustrate this concept, Chen et al. designed two kinds of prodrugs fabricated with NLG919 and Fmoc, conjugated to polyethylene glycol (PEG). PEG_2k_‐Fmoc‐NLG (L) contained the more labile linker, while PEG_2k_‐Fmoc‐NLG(S) contained the stable amide linker as a control group (**Figure** **6**).^[^ ^103^ ^]^ When the two conjugates self‐assemble, the prodrug nanoparticles are formed, and a chemotherapeutic drug (PTX) can be physically encapsulated inside. In addition, the Fmoc group in the dual‐functional prodrug can improve drug loading and formulation stability.^[^ ^104^ ^]^ This nanosystem can simultaneously deliver both drug components (NLG919 and PTX) to the tumor by chemical bonding and physical encapsulation, respectively. Compared with the stable (S) group, the labile (L) group showed a faster release of NLG919 with a more significant antitumor activity, though enhancing the T‐cell immune response. Further, the amount of CD8^+^ and CD4^+^ T cells in the tumor were increased after treatment with PEG_2k_‐Fmoc‐NLG(L).

**Figure 7:**
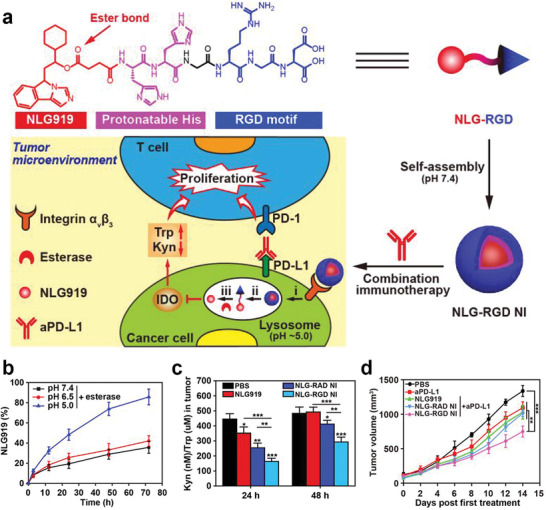
a) Self‐assembly of peptide‐drug conjugate and the combination immunotherapy mechanism of NLG‐RGD NI and aPD‐L1. b) NLG919 release from NLG‐RGD NI treatment at different pH plus esterase. c) Kyn/Trp ratio in tumor from mice treated with different formulations. d) Tumor growth curve of tumor‐bearing mice with different formulation. Reproduced with permission.^[^ ^105^ ^]^ Copyright 2020, American Chemical Society.

Along with the PEGylated IDO inhibitor, Han et al. recently constructed a peptide‐conjugated NLG919 prodrug for reducing IDO‐1‐induced immune resistance.^[^ ^105^ ^]^ First, the authors synthesized a peptide‐drug conjugate by conjugating the arginyl‐glycyl‐aspartic acid (RGD) with NLG919, a potent IDO‐1 inhibitor via two protonatable histidine, which self‐assembled into the IDO nanoinhibitors as the peptide portion for inhibiting nanoparticle aggregation, and also provided water stability. The NLG919 prodrug showed some significant advantages, including good tumor‐homing ability via modification of RGD, pH‐responsive disassembly by protonatable histidine, and controllable drug release of NLG919 though esterase‐catalyzed hydrolysis (**Figure** **7**a,b). The authors discovered that the NLG919 prodrug had reduced systemic toxicity and better inhibition of intratumoral IDO compared with free NLG919 and RGD‐free NLG919 prodrugs, due to improved tumor accumulation and controlled release of NLG919 (Figure 7c). Furthermore, the combination of IDO inhibition and PD‐L1 blockade displayed improved therapeutic efficacy by addressing the disadvantages of monotherapy with a single immune checkpoint inhibitor (Figure 7d).

### 2.5 Immune Checkpoint Inhibitor‐Based Prodrug Nanomedicines for Attenuating Immune Tolerance

In past few years, monoclonal antibody‐based ICB therapy has remarkably advanced cancer immunotherapies in the clinic.^[^ ^106^ ^]^ Nevertheless, the antibodies can cause severe side effects: 1) the targets of the immunological checkpoint antibodies are not only distributed on tumor infiltrating lymphocytes, tumor‐associated macrophages, and tumor cells, but are also widely distributed in normal tissues. Immune cells can then attack important organs such as the kidneys, potentially resulting in autoimmune diseases;^[^ ^107^ ^]^ 2) antibody drugs have a long circulation half‐life in vivo, prolonging the duration and intensity of action. However, this long‐circulating ability also causes a large accumulation of antibodies in nontarget tissues, resulting in toxic side effects.^[^ ^108^ ^]^ Therefore, the distribution characteristics of immune checkpoint inhibitors such as “on‐target, but off‐tumor”^[^ ^109^ ^]^ should also be considered in development of antibody treatments.^[^ ^110^ ^]^ Fortunately, nanoparticles have improved tumor‐targeting ability due to the EPR effect. Hence, the deficiency of antibodies could be ameliorated by conjugating the antibody to nanoparticles, tumor immune cells, etc.

**Figure 8:**
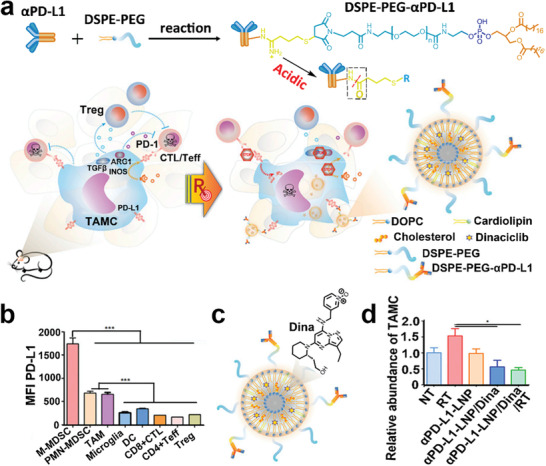
a) Schematic illustration of *α*PD‐L1‐LNPs and RT to kill TAMCs and against immunosuppression. b) The expression level of PD‐L1 on different immune‐related cells. c) chemical structure of Dina and schematic of *α*PD‐L1‐LNPs loaded with Dina. d) FACS analysis of abundance of TAMCs from GL261‐bearing mice treated with different formulations. Reproduced with permission.^[^ ^112^ ^]^ Copyright 2019, the National Academy of Sciences of USA.

Liposomes represent the most widely used nanoformulations for clinical cancer treatment.^[^ ^111^ ^]^ Hence, the modification of liposome with the antibodies is a promising nanoimmunotherapy approach. Tumor‐associated myeloid cells (TAMCs) are an important contributor to immunosuppression, and are dramatically recruited in glioblastoma, where they express more PD‐L1 than tumors as well as other immune cells. To target TAMCs, Zhang et al. developed an *α*PD‐L1‐functionalized lipid nanoparticle (*α*PD‐L1‐LNPs) for combination immunotherapy with radiotherapy (RT).^[^ ^112^ ^]^ The *α*PD‐L1‐LNPs loaded with a cyclin‐dependent kinase inhibitor (dinaciclib) improved localized delivery of dinaciclib (Dina) to TAMCs by recognizing PD‐L1 expressed on the surface of TAMCs and led to the depletion of the TAMCs and an impressive attenuation of immunosuppression (**Figure** **8**a,b). The *α*PD‐L1‐LNPs loaded with Dina showed comparable cytotoxicity against TAMCs in comparison with free Dina, indicating that *α*PD‐L1‐LNPs had a high drug efficiency (Figure 8c). The combination of *α*PD‐L1‐LNPs plus RT showed an excellent ability to target TAMC, as the expression of PD‐L1 on the surface of glioma‐associated TAMCs was dramatically upregulated after RT. The combination of *α*PD‐L1‐LNPs encapsulating Dina and laser irradiation significantly reduced local immunosuppression and enhanced the apoptosis of glioma cells, resulting in prolonged survival in two models of glioma (Figure 8d).

In addition to the lipid components of nanoparticles the FDA‐approved biodegradable polymer poly(lactic‐*co*‐glycolic‐acid) (PLGA) is also employed for cancer immunotherapy.^[^ ^113^ ^]^ The hydrophobic core of PLGA NPs can be loaded with various types of payloads to prevent their degradation or premature leakage during circulation in blood. To achieve tumor‐targeted delivery of PLGA nanoparticles carrying immune modulators, Schmid et al. conjugated maleimide‐functionalized PEG‐PLGA nanoparticles with Immunoglobulin G (IgG) antibody fragments for codelivery of dual immunomodulatory regimens. The antibody‐functionalized nanoparticles specifically adhered to the surface of T cells by recognizing the receptors on the surface of the T cell membrane,^[^ ^114^ ^]^ and the loaded drugs displayed a sustained release from the T cell‐targeting PLGA NPs. The T cell‐targeting PLGA NPs, loaded with TGF‐*β*1 inhibitor (SD‐208),^[^ ^115^ ^]^ suppressed tumor growth and prolonged the survival of tumor‐bearing mice by promoting T cell proliferation and inhibiting TGF‐*β*1 activity.

**Figure 9:**
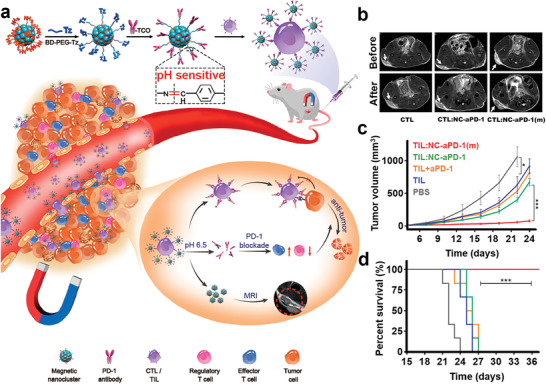
a) Schematic illustration of the pH‐responsive PD‐1 antibody conjugated magnetic nanoclusters to reverse the tumor immunosuppressive and achieve high antitumor efficiency. b) T2‐MR imaging of mice before and after receiving different CTL formations. c) Tumor growth curves and d) survival curves of tumor‐bearing mice with different treatments. Reproduced with permission.^[^ ^120^ ^]^ Copyright 2019, American Chemical Society.

Magnetic nanoclusters (NCs) have also recently been utilized for magnetic resonance imaging (MRI) and drug delivery.^[^ ^116^ ^]^ In comparison with polymeric or liposomal NPs, magnetic NCs possess several distinct advantages: 1) multiple functions can be integrated into NCs through the use of surface modifications; 2) nanoparticle trafficking can be easily visualized by magnetic resonance imaging (MRI);^[^ ^117^ ^]^ 3) magnetic NCs can be specifically guided to the tumor site by super paramagnetism.^[^ ^118^ ^]^ To take advantage of these characteristics of magnetic NCs, Nie et al. developed *α*PD‐1 antibody (*α*PD‐1)‐conjugated magnetic NCs via the inverse electron demand Diels‐Alder reaction and incorporation of a pH‐sensitive benzoic‐imine bond.^[^ ^119^ ^]^ The resulting NCs‐aP can specifically interact with effector T cells overexpressing PD‐1 (**Figure** **9**a).^[^ ^120^ ^]^ Under the guidance of MRI, the NCs‐aP facilitated the intratumoral infiltration of effector T cells at tumor sites (Figure 9b). The authors found that NCs‐*α*PD‐1 could locally release *α*PD‐1 after cleavage of the benzoimine bond in the acidic intratumoral microenvironment. The released *α*PD‐1 suppressed tumor growth and metastasis by reversing the immunosuppressive effect in the tumor microenvironment, and was reported to have high efficiency and low toxicity (Figure 9c,d).

**Figure 10:**
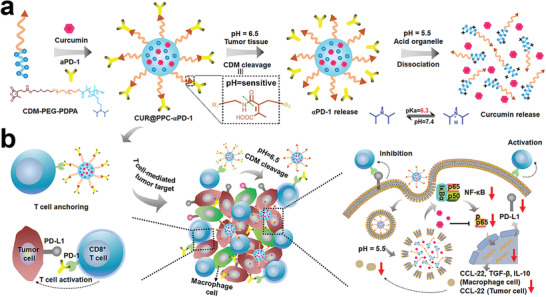
a) Preparation of dual pH‐sensitive nanomedicine and the sequentially release mechanism of nanomedicine upon different pHs. b) Tumor‐targeted delivery of nanomedicine by modification of *α*PD‐1 and enhancement of CUR into tumor cells for synergistic therapy of PD‐1 blockade and NF‐*κ*B inhibition. Reproduced with permission.^[^ ^122^ ^]^ Copyright 2019, American Association for the Advancement of Science (AAAS).

Several recent lines of evidence have suggested that the ITM is simultaneously regulated by multiple immune evasion mechanisms. Among these, the nuclear factor kappa B (NF‐*κ*B) signaling pathway, which promotes the proliferation of Tregs, has attracted extensive attention.^[^ ^121^ ^]^ Therefore, the combination of NF‐*κ*B inhibition and aforementioned PD‐1 blockade could represent a potential strategy for reversing multiple mechanisms of immune evasion. Nevertheless, this strategy faces formidable challenges associated with improving the codelivery of multiple immune modulators. To address this dilemma, Xiao et al. designed dual pH‐sensitive prodrug nanoparticles that were decorated with PD‐1 antibodies (*α*PD‐1) on their surface via an ammonolysis reaction and were loaded with curcumin (CUR) in their core (**Figure** **10**).^[^ ^122^ ^]^ The *α*PD‐1‐modified nanoparticles specifically adhered to the circulating PD‐1^+^ T cells by recognizing the PD‐1 expressed on the surface of T cell membrane, leading to effective tumor accumulation with the help of T cells. Afterward, the aPD‐1 modified nanoparticles specifically released *α*PD‐1 at the tumor site due to cleavage of the pH‐sensitive linker in the acidic tumor microenvironment. There, the surface charge was changed from negative to positive, enhancing PD‐1 blockade on T cells by *α*PD‐1 and also promoting uptake of the nanoparticles by tumor cells. After being endocytosed into the lysosomes of tumor cells, the pH‐responsive nanomedicine disassembled, resulting in rapid release of the encapsulated CUR. The CUR inhibited the NF‐*κ*B pathway, enhancing the effect of PD‐1/PD‐L1 immunotherapy. This combination led to simultaneous blockade of the PD‐1/PD‐L1 pathway and reduction of the immunosuppressive effects caused by the Tregs, resulting in impressive antitumor efficiency.

**Figure 11:**
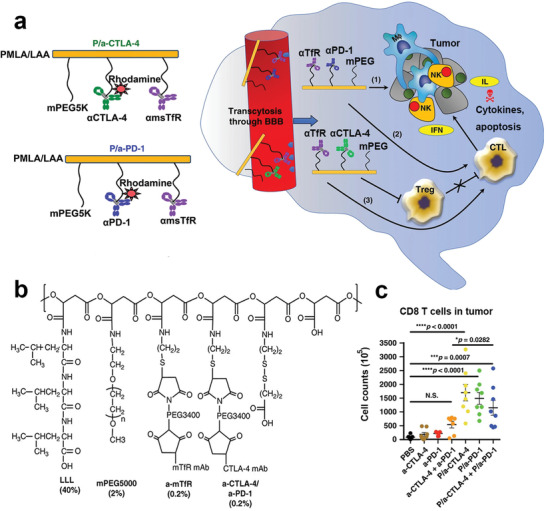
a) Schematic illustration and b) chemical structure of modified PMLA/LLL conjugated with different antibodies. c) Polymeric prodrug of *α*PD‐1 and *α*CTLA‐4 for glioblastoma therapy. d) FACS analysis of CD8^+^ T cells from brain tumors receiving with different formulations. Reproduced with permission.^[^ ^125^ ^]^ Copyright 2019, Springer Nature.

In recent years, the combination of *α*CTLA‐4 antibody and *α*PD‐1 for ICB therapy has yielded much better clinical benefits than monotherapy due to significant activation of CTLs and suppression of Tregs.^[^ ^123^ ^]^ However, the combination of immune checkpoint inhibitor does not work well for brain tumors such as gliomas since the blood–brain barrier (BBB) prevents antibodies from entering the brain.^[^ ^124^ ^]^ To address this challenge, Galstyan et al. developed a novel antiglioblastoma immunotherapy for local activation of antitumor immune response in the brain by a natural biopolymer scaffold (PMLA/LLL) modified with *α*PD‐1 or *α*CTLA‐4 linked to nanoscale immunoconjugates (NICs) (**Figure** **11**).^[^ ^125^ ^]^ The NICs efficiently cross the BBB after conjugation with anti‐TfR or angiopep‐2 peptide, and selectively release the antibodies at the tumor site by ROS‐mediated cleavage of the thioether bond. The glioblastoma was thus significantly inhibited after treatment with the multifunctional NIC due to recruitment of CTLs and suppression of Tregs.

Exosomes, a natural nanoscale drug delivery platform, have some advantages over synthetic nanoplatforms, such as low immunogenicity and good biocompatibility. As the exosomes contain some specific contents from the produced cells, they retain the functions and physiological features of the original cells.^[^ ^126^ ^]^ Therefore, immune cell‐derived exosomes could play a great role in immunotherapy.^[^ ^127^ ^]^ For example, DC‐derived exosomes can stimulate T‐cell immune responses.^[^ ^128^ ^]^ In contrast, the exosomes extracted from M1 macrophages can reprogram M2 type macrophages to M1 macrophages to reverse ITM.^[^ ^129^ ^]^ The exosomes can be further modified with targeting ligands for selective delivery of immune modulators. For instance, Nie et al. functionalized an M1 macrophage‐derived exosome nanoplatform with antisignal‐regulatory protein *α* antibody (*α*SIRP*α*) and *α*CD47 using a pH‐responsive benzoic‐imine linker.^[^ ^130^ ^]^ The *α*CD47‐conjugated M1 exosome displayed an impressive tumor‐targeting ability by specifically recognizing CD47 expressed on the surface of tumor cells. After reaching the acidic tumor microenvironment, the conjugated *α*CD47 and *α*SIRP*α* were locally released from the nanoplatform by cleavage of the acid‐labile bond. The released *α*CD47 suppresses the CD47, which otherwise causes immune tolerance, and the released *α*SIRP*α* reactivates macrophages by inhibiting SIRP*α*, ultimately enhancing phagocytosis. More importantly, the native M1 exosomes could increase the content of antitumor M1 macrophages by reformatting the protumor M2 macrophages.

### 2.6 Binary Cooperative Prodrug Nanomedicines for Improved Cancer Immunotherapy

The antitumor immunity induced by chemotherapeutics is often insufficient for clinical response. Thus, there is a strong need to combine the core strengths of chemotherapeutics with other strategies to synergistically enhance antitumor immune responses. Photodynamic therapy (PDT) is one such strategy that can enhance antitumor immunity by inducing ICD.^[^ ^131^ ^]^ The principle of PDT is that a specific wavelength of light is locally irradiated to the accumulated photosensitizer (PS) at the tumor site, and the activated PS generates cytotoxic singlet oxygen to cause tumor cell ablation and ICD.^[^ ^132^ ^]^ Unfortunately, PDT‐based cancer immunotherapy is challenged by the poor water solubility and severe phototoxicity of PSs.

**Figure 12:**
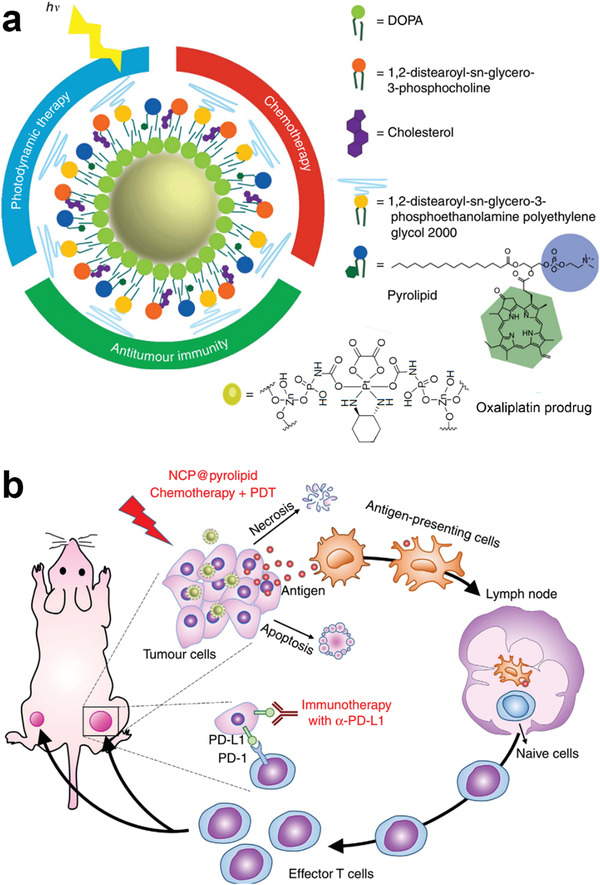
a) Schematic illustration of the therapeutic modalities and component structure of NCP@pyrolipid prodrug NPs; b) NCP@pyrolipid‐mediated combination of three therapeutic modalities for triggering ICD of the tumor cells and blockading PD‐1/PD‐L1 pathway. Reproduced with permission.^[^ ^133^ ^]^ Copyright 2016, Springer Nature.

Conjugation of PSs and phospholipids has been tested as a means to improve the efficiency of PDT generated by PSs. The modified phospholipid can not only be used as a component of the liposome, but also increases the drug loading of the PSs. To this end, He et al. developed core–shell nanoscale synergistic vesicles (called NCP@pyrolipid) that were combined with *α*PD‐L1 therapy for tumor immunotherapy. This nanoplatform integrated three therapeutic modalities: chemotherapy, PDT, and immunotherapy.^[^ ^133^ ^]^ The core of the NCP@pyrolipid consisted of an OXA prodrug. The released oxaliplatin led to chemotherapy and also induce ICD. The shell of the (NCP@pyrolipid) contained a pyropheophorbide‐lipid conjugate (pyrolipid), which induced ICD by PDT (**Figure** **12**a).^[^ ^46^, ^134^ ^]^ The authors demonstrated comparable blood circulation half‐life (11.8 ± 1.9 h vs 8.4 ± 2.6 h) of OXA and NCP@pyrolipid, indicating a similar pharmacokinetic profile of free OXA and the nanoparticle formulation of the OXA prodrug. The combination of PD‐L1 blockade and NCP@pyrolipid‐based PDT efficiently promoted the secretion of IFN‐*γ* and increased the intratumoral infiltration of CTLs, resulting in inhibition of growth of both the primary and metastasized tumors (Figure 3b).^[^ ^133^ ^]^

**Figure 13:**
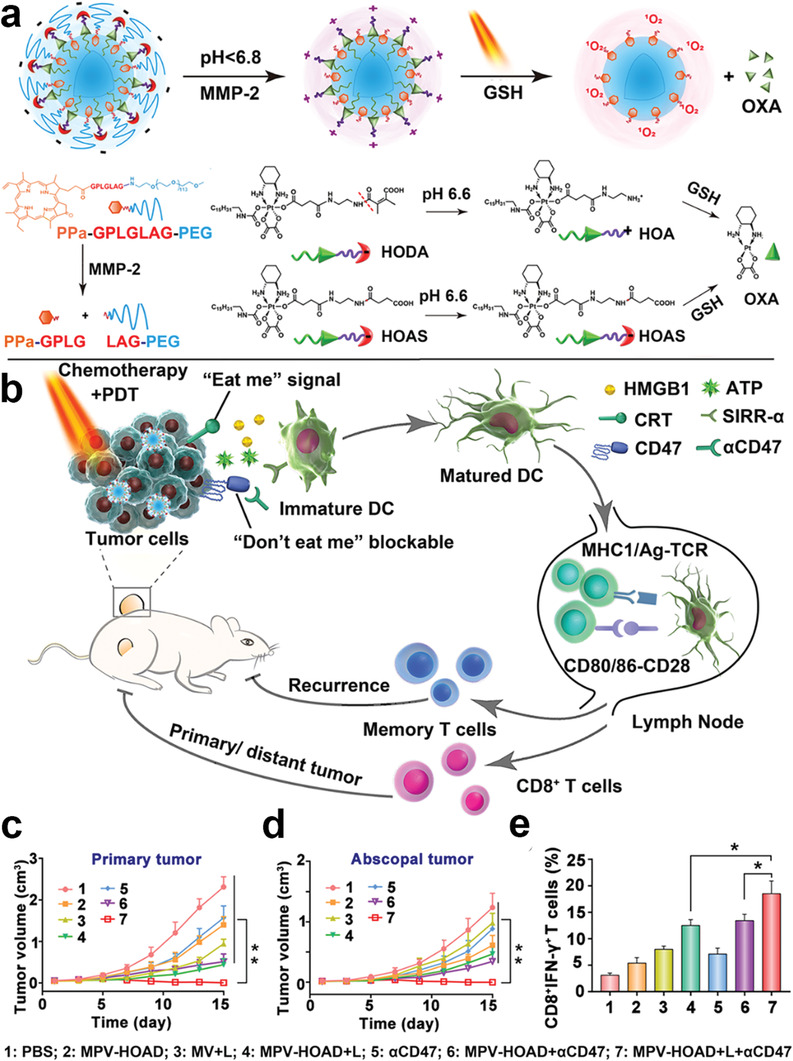
Schematic illustration of multiresponsive prodrug a) vesicles and b) prodrugs. Relative c) primary and d) abscopal tumors growth curve of tumor‐bearing mice with different treatments. Frequency of e) CD8^+^/IFN‐*γ* ^+^ T cells. Reproduced with permission.^[^ ^137^ ^]^ Copyright 2019, John Wiley & Sons.

Recently, several studies have revealed that the aggregation‐caused quenching (ACQ) effect of PS impairs the therapeutic efficacy of PDT by suppressing ROS generation.^[^ ^16^, ^135^ ^]^ To minimize this ACQ effect, extensive efforts have aimed to develop a tumor microenvironment‐responsive PS prodrug. The PS prodrug forms aggregates in the systemic circulation, which helps reduce phototoxicity before reaching the tumor site.^[^ ^136^ ^]^ The PS prodrug increases the yield of ROS by cleaving the responsive linker to release the PS only at the tumor site. In turn, increased ROS production promotes ICD, enhancing the immune response. For example, Zhou et al. constructed tumor microenvironment‐activatable prodrug vesicles combined with *α*CD47 that prevented tumor metastasis and recurrence by ICD and CD47 blockade. The prodrug vesicles were composed of a dual‐sensitive (pH and GSH) oxaliplatin prodrug (HODA) and a MMP‐2 responsive PEGylated photosensitizer prodrug (PPa‐GPLGLAG‐PEG) (**Figure** **13**).^[^ ^137^ ^]^ Upon 671 nm laser irradiation, the prodrug vesicles displayed a laser‐triggered drug release profile that led to a hyperthermic effect. Furthermore, the prodrug vesicles showed an elongated Pt clearance half‐time compared with free OXA in vivo. The prodrug vesicles also displayed improved tumor accumulation and penetration due to the acid‐activated charge reversal and MMP‐2‐sensitive de‐PEGylation. Many tumor cells overexpress immune checkpoint CD47,^[^ ^138^ ^]^ a membrane‐bound protein that prevents phagocytosis of tumor cells by DCs and macrophages.^[^ ^139^ ^]^ Mice treated with the prodrug vesicles and *α*CD47 had less tumor microenvironment immunosuppression as indicated by decreased proliferation of Treg cells and increased tumor‐infiltrating CTLs, leading to less tumor metastasis and tumor recurrence.^[^ ^137^ ^]^

Cancer cells and their microenvironments could establish enormous negative feedback mechanisms to evade the surveillance of the immune system. For instance, the aforementioned IDO could suppresses the activity of the effector T cells via regulating the degradation of tryptophan, and PD‐L1/PD‐1 pathway leads to the exhaustion of T cells after the recognition after the recognition of T cells with tumor cells. Hence, the synergetic therapeutic strategy to simultaneously inhibit the PD‐L1/PD‐1 pathway and IDO pathway could be an ideal choice for effectively relieving the immunosuppressive TME, leading to an enhanced cancer immunotherapy. What's more, the use of antibodies to block interaction between PD‐L1/PD‐1 is currently the prevailing strategy in clinical practice.^[^ ^140^ ^]^ Despite advances with antibody drugs, there are still some shortcomings, including poor stability, relatively high production costs, significant immunogenicity, and low efficiency in penetrating tumors. Hence, at present, there is an opportunity for the development of low molecular weight antagonists of immune checkpoint to block the PD‐1/PD‐L1 axis. Peptide‐based immune modulators represent an attractive alternative due to their higher stability, lower cost, lower immunogenicity, and excellent tumor penetration.^[^ ^141^ ^]^

**Figure 14:**
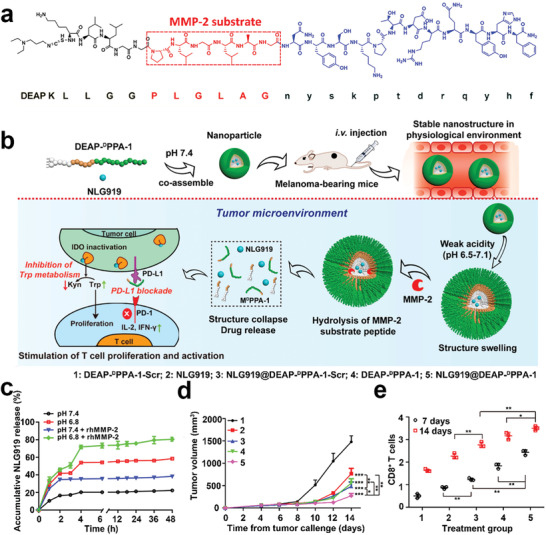
a) Amino acid composition of hydrophobic domain (DEAP), MMP‐2 substrate and hydrophilic domain (DPPA‐1). b) Mechanism of the self‐assembled NLG919@DEAP‐DPPA‐1 for precisely controlling drug release and enhancing immunotherapy: 1) nanostructure could remain stable at physiological environment; 2) the hydrophobic core swells after penetrating into the acidic tumor stroma; 3) MMP‐2 hydrolyze the MMP‐2 responsive peptide; 4) nanoparticles collapse to localized release of NLG919 and DPPA‐1 peptide for blockading the IDO and PD‐L1 pathways. c) NLG919 release profiles of NLG919@DEAP‐DPPA‐1 under different conditions. d) Tumor growth curve of tumor receiving different formulations. e) Flow cytometric quantification of CD8^+^ T cells after different treatment. Reproduced with permission.^[^ ^143^ ^]^ Copyright 2018, American Chemical Society.

Given above advantages of peptide‐based immune antagonists, Cheng et al. developed an amphiphilic therapeutic peptide prodrug consisting of a responsive hydrophobic domain and a therapeutic peptide hydrophilic domain. The hydrophobic domain was based on a conjugate of a MMP‐2‐responsive peptide substrate^[^ ^142^ ^]^ with 3‐diethylaminopropyl isothiocyanate (DEAP), the hydrophilic domain contained a PD‐1 antagonist peptide (^D^PPA‐1) (**Figure** **14**).^[^ ^143^ ^]^ The hydrophilic domain contained a PD‐1 antagonist peptide (^D^PPA‐1). The amphiphilic peptide prodrug could self‐assemble into a nanocarrier and encapsulate IDO inhibitors (NLG919) into a hydrophobic core. Upon encountering the weakly acidic microenvironment at the tumor site, the nanostructures swell due to protonation of the DEAP molecule and then collapse after cleavage of the PLGLAG spacer by MMP‐2, which is overexpressed in the tumor stroma. The cleavage of the prodrug and collapse of the nanoparticles leads to a steady codelivery (and precisely controllable release) of NLG919 and PD‐1 antagonist. The combination of a PD‐1 antagonist and NLG919 efficiently blocked both the PD‐L1/PD‐1 and IDO‐1 pathways, restoring the antitumor activity of CTLs to prevent tumor regression.

## 3 Binary Cooperative Prodrug Nanomedicines for Immune Normalization Therapy

**Figure 15:**
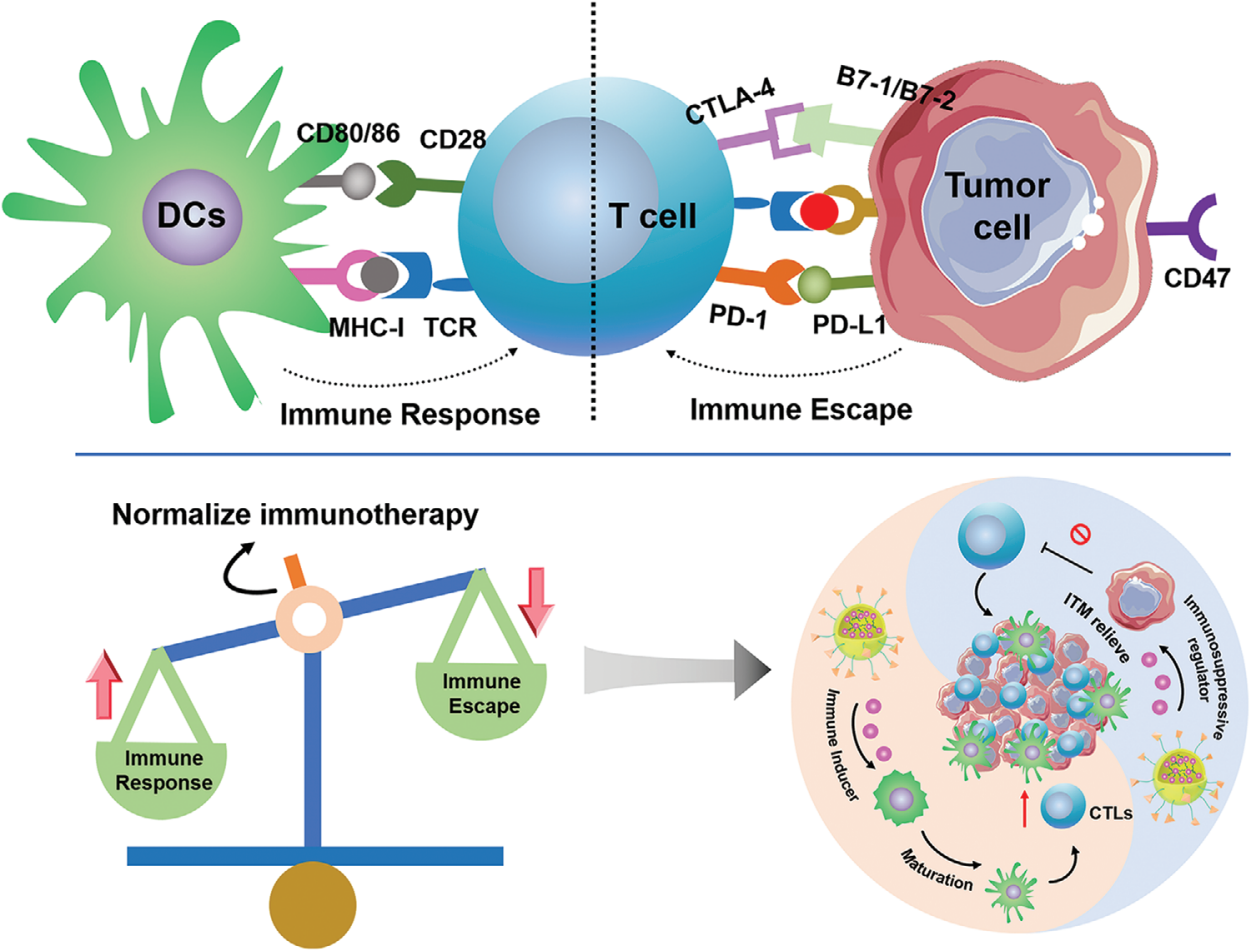
Schematic illustration of adaptive immune response axis for cancer immunotherapy, the combination therapy strategy for immune normalization.

Current immunotherapy hits a stumbling block due to insufficient immunogenicity of the tumor cells and ITM. Monotherapy either by eliciting antitumor immune response or reversing the ITM often has a discount effect. In view of this situation, there is an urgent need for combination‐therapy strategies to improve the effects of immunotherapy by promoting immune responses and preventing immunosuppression (**Figure** **15**). In recent years, a variety of combination immunotherapy treatments have been investigated, such as PDT combined with immune checkpoint blockers, chemotherapy combined with small molecule immune inhibitors, and even tri‐therapies (**Table** **2**). Nevertheless, there remains uncertainty on how to optimize the combined therapies, due to complex regulatory interactions between various molecules and the potential for systemic side effects. Here, nanomedicine offers advantages in facilitating the codelivery of multiple drugs and precise tuning of drug release.

**Table 2:** Prodrug nanomedicine synergistically boosting cancer immunotherapy

Main type	Active component in prodrug	Delivery platform	Responsive condition	Assisted ingredient	Immunological effects	Refs.
PDT and immunosuppressive inhibitor	PPa, NLG919	Host–guest supramolecular prodrug nanocomplexes	GSH	**–**	Enhance the immune response and reduce the immunosuppress	^[^ ^147^ ^]^
	OSPS, NLG919	Organic semiconducting pro‐nanostimulant (OSPS) nanoplatforms	ROS	–	Enhance the immune response and reduce the immunosuppress	^[^ ^153^ ^]^
	PpIX, IDO inhibitor (1MT)	Small molecular weight prodrug nanoparticles	Caspase 3	–	Enhance the immune response and reduce the immunosuppress	^[^ ^156^ ^]^
	PPa, NLG919	Sheddable prodrug vesicles	MMP‐2, GSH	–	Enhance the immune response and reduce the immunosuppress	^[^ ^157^ ^]^
	PPa, NLG919	Boolean logic prodrug nanoparticle	MMP‐2, GSH, pH	–	Enhance the immune response and reduce the immunosuppress	^[^ ^158^ ^]^
Chemotherapy and immunosuppressive inhibitor	SN‐38, indomethacin	Prodrug conjugate	Redox	–	Reduce the production of TNF‐*α* and IL‐6 and enhance the production of IL‐10	^[^ ^165^ ^]^
	OXA, NLG919	Binary cooperative prodrug nanoparticle	Reduction	–	Enhance ICD‐associated immunogenicity and alleviate the tumor microenvironment immunosuppression	^[^ ^23^ ^]^
	OXA, NLG919, PPa	Multifunctional prodrug nanoplatform	ROS, GSH	**–**	Enhance ICD‐associated immunogenicity and alleviate the tumor microenvironment immunosuppression	^[^ ^168^ ^]^

### 3.1 Prodrug Nanomedicine for Combination PDT and IDO‐Blockade Therapy

As mentioned above, PDT not only induces tumor cell apoptosis and/or necrosis by inducing ROS generation, but also causes ICD of the tumor cells and, subsequently, initiates an antitumor immune response. However, there are several factors resulting in strong local immunosuppression, including IDO,^[^ ^144^ ^]^ interleukin 10 (IL‐10),^[^ ^145^ ^]^ TGF‐*β*,^[^ ^146^ ^]^ PD‐1/PD‐L1 pathway, CD47, and so on. Thus, there is a general need for a strategy using inhibitors to block these immunosuppressive factors. An IDO pathway inhibitor (NLG919) has shown encouraging clinical outcomes. However, commonly used small‐molecule drugs such as PSs and IDO inhibitors have shown poor effects when administered in free form. Therefore, modification of small‐molecule drugs into prodrugs using tumor‐response linkages could both avoid the premature leakage of IDO inhibitors and rapid clearance of PSs from circulation. Relatedly, some heterodimers of hydrophobic prodrugs cannot self‐assemble into stable nanomedicines without the assistance of amphiphilic surfactant molecules.

**Figure 16:**
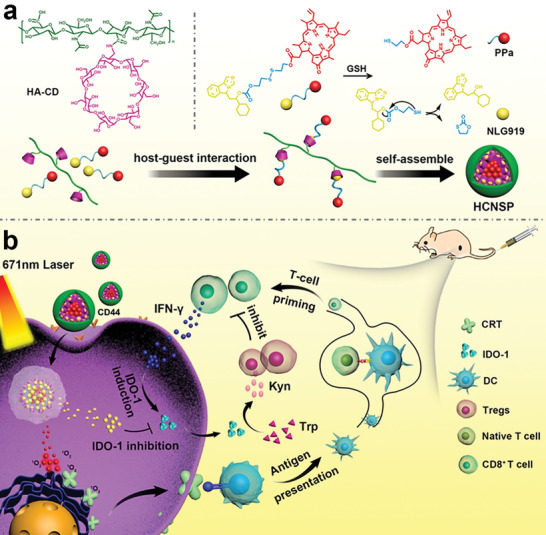
Upper: Preparation of supramolecular prodrug nanocomplexes based on the host–guest interaction of HA and heterodimer prodrug; bottom: mechanism of achieving combination immunotherapy via ROS‐mediated immunogenicity and NLG919‐mediated IDO‐1 inhibition. Reproduced with permission.^[^ ^147^ ^]^Copyright 2020, John Wiley & Sons.

To deal with this dilemma, Hu et al. developed supramolecular prodrug nanocomplexes aimed to codeliver two different immune modulators to combat low tumor immunogenicity and the immunosuppressive TME (**Figure** **16**).^[^ ^147^ ^]^ Through this host‐guest interaction, hyaluronic acid (HA) and the synthesized glutathione‐triggered dimer of NLG919 and pheophorbide could be self‐assembly into supramolecular prodrug nanocomplexes. These showed excellent tumor‐targeting ability due to the HA–CD44 interaction. In addition, this heterodimer prodrug allows for precise control over the release of NLG919 and pheophorbide through making use of the highly reducing environment at the tumor site. More importantly, the host–guest nanoplatform showed an effective combination immunotherapy, acting by causing ROS‐mediated immunogenicity and NLG919‐mediated inhibition of IDO‐1.

Despite its significant enthusiasm within the field, PDT has many shortcomings,^[^ ^148^ ^]^ including photobleaching of photosensitizers, serious tumor hypoxia, and poor penetration of near infrared (NIR) light, which together contribute to high chances of regrowth of residual tumor cells.^[^ ^149^ ^]^ However, a new class of organic photothermal nanomaterials transformed from optically active semiconductor polymers have displayed excellent PDT and photothermal therapy (PTT) properties.^[^ ^150^ ^]^ Compared to inorganic nanomaterials, these exhibit good biocompatibility and excellent optical properties.^[^ ^151^ ^]^ In addition, several groups discovered that both PDT and PTT can induce immunogenic cell death, leading to increased immune responses.^[^ ^152^ ^]^ For example, Li et al. reported near‐infrared photoactivatable organic semiconducting pro‐nanostimulant nanoplatforms (SPNs) by integrating organic semiconducting pro‐nanostimulant (OSPS) and the IDO‐1 inhibitor NLG919.^[^ ^153^ ^]^ The authors demonstrated that the OSPS, under laser irradiation, produces both heat and ^1^O_2_, not only generating tumor‐associated antigens, but also cleaving the ^1^O_2_‐liable linkers. Furthermore, NLG919 conjugated to the OSPS via the ^1^O_2_‐cleavable linkers led to near‐infrared photoactivatable release, significantly reducing the risk of immune‐related adverse events (IRAEs). Subsequently, the authors revealed that SPNs could both suppress the growth of primary/distant tumors and prevent lung metastasis via the combination of PDT/PTT and NLG919.^[^ ^154^ ^]^

**Figure 17:**
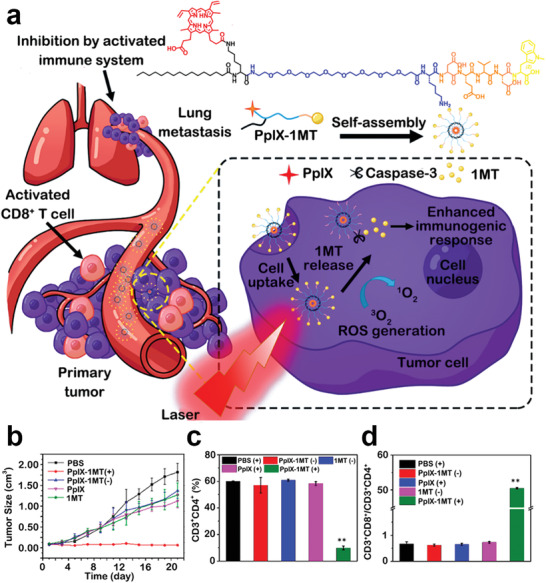
a) Preparation of PpIX‐1MT nanoparticles and the mechanism of enhanced immunogenic response. b) Tumor growth of mice treated with different formulations. c) The treatment‐induced activation of CD4^+^ T and d) CD8^+^ T cells in the tumors. Reproduced with permission.^[^ ^156^ ^]^ Copyright 2018, American Chemical Society.

Another widely used IDO inhibitor is dextro‐1‐methyl tryptophan (referred to as D‐1MT), which can prevent from degrading the activity of T cell triggered by IDO via reducing tryptophan catalyzed by kynurenine.^[^ ^155^ ^]^ Combining the advantages of low molecular weight prodrugs and nanocarriers into consideration, Song et al. designed a caspase‐activatable, multifunctional, prodrug nanoplatform for inhibiting cancer metastasis and recurrence.^[^ ^156^ ^]^ The caspase‐responsive multifunctional prodrug (PpIX‐1MT) was synthesized by a caspase‐responsive peptide sequence modified with IDO inhibitor (1MT) and photosensitizer (PpIX), and the PpIX‐1MT self‐assembled into nanoparticles without the addition of any other excipients (**Figure** **17**a). PpIX‐1MT NPs displayed a long blood circulation profile and tumor specific distribution in tumor‐bearing mice. Upon laser irradiation, the PpIX‐1MT NPs triggered a cascading, synergistic effect, in which i) PDT generates ROS to cause apoptosis of cancer cells, ii) the apoptosis of cancer cells produces tumor antigens and expression of caspase‐3, iii) expression of caspase‐3 cleaves the PpIX‐1MT to release 1MT, iv) 1MT initiates the immune response and recruits CTLs, and v) the combination of PDT and immunotherapy suppresses tumor metastasis and recurrence. Upon light irradiation, compared with the tumor‐bearing mice treated with free 1MT and free PpIX, as well as PpIX‐1MT NPs without laser, the PpIX‐1MT NP‐treated mice not only showed significantly inhibited tumor growth, but also increased activation of CD8^+^ T lymphocytes due to IDO blockade (Figure 17b–d).

**Figure 18:**
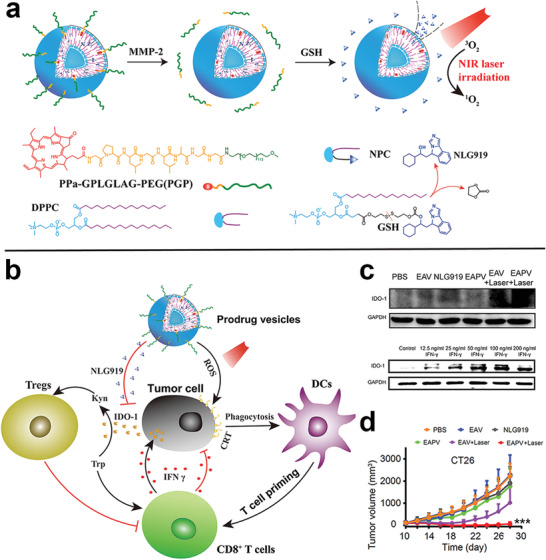
a) The dual‐responsive (acidic and MMP‐2) mechanism of sheddable prodrug vesicles and prodrugs. b) Schematic illustration of combined therapeutic modalities to increase ICD and blockading IDO pathway. c) Expression of IDO‐1 in CT26 tumors and IFN‐*γ*‐induced IDO‐1 expression in CT26 cells analyzed by western‐blotting. d) Tumor growth of CT26 mice treated with different formulations. Reproduced with permission.^[^ ^157^ ^]^ Copyright 2019, American Chemical Society.

Solid tumors have dense extracellular matrices and high fluid pressure, which reduces the deep penetration and accumulation of NPs in tumors. Modification with PEG can dramatically enhance particle circulation kinetics (thus increasing tumor diffusion through the EPR effect), but also reduces the cellular uptake of nanoparticles, producing the so‐called PEG dilemma. To address this disadvantage of conventional NPs, Gao et al. constructed sheddable prodrug nanovesicles that can both promote deep tumor penetration and improve cancer immunotherapy (**Figure** **18**).^[^ ^157^ ^]^ Two prodrugs of NLG919 and PPa were synthesized, respectively. The PPa served as a PS and was conjugated with PEG via a MMP2‐sensitive peptide spacer, and NLG919 was coupled with phospholipid via a disulfide bond. The authors reported that the sheddable prodrug vesicles showed an excellent MMP‐2 and GSH dual‐responsive ability in vitro and better plasma stability compared with free NLG919 in vivo. In addition, the combination therapy with PDT and inhibition of IDO both enhanced the immune response by inducing ICD and also reduced immunosuppression by inhibiting tryptophan degradation.

**Figure 19:**
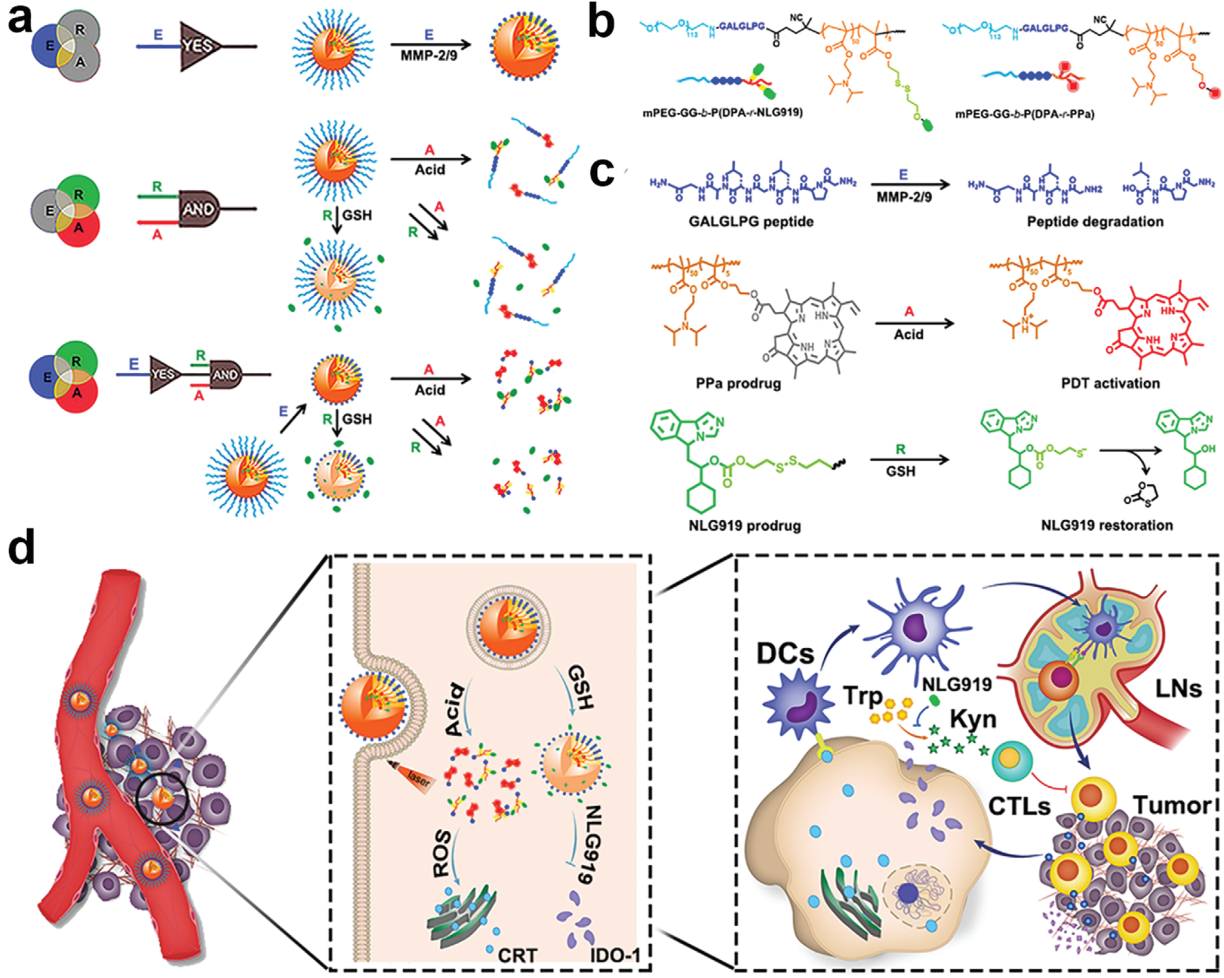
a) Mechanisms of BLPNs for cancer immunotherapy by utilizing Boolean logic. b) Chemical structures and c) degradation pathways of two stimuli‐activatable polymeric prodrugs. d) Schematic illustration of BLPNs for cancer immunotherapy by enhancing antitumor immunogenicity and IDO‐1 inhibition. Reproduced with permission.^[^ ^158^ ^]^ Copyright 2020, John Wiley & Sons.

To further achieve spatiotemporally controllable drug delivery at the tumor site, Hou et al. designed a Boolean logic prodrug nanoparticle (BLPN) to provide precise immunotherapy of cancer. BLPNs were composed of two stimuli‐activatable polymeric prodrugs to realize ultraprecise codelivery of different immune modulators into the tumor and achieve sequential stimuli‐activatable release (**Figure** **19**a).^[^ ^158^ ^]^ In this article, one polymeric prodrug was fabricated by conjugating the immune inhibitor of NLG919 to the protonatable polymer chain via a reduction‐sensitive disulfide bond, and another polymeric prodrug was synthesized by grafting the immune activator of pheophorbide a to the same protonatable polymer chain via a labile ester bond. Further, both of the protonatable polymer chains contained an enzymatically degradable peptide sequence, and the two polymeric prodrugs actually self‐assemble into nanoparticles, leading to no activity in systemic circulation (Figure 19b,c). Afterward, the authors demonstrated that the BLPNs could perform selective and tunable logic operations to dissociate out of being nanoparticles and activate the polymer prodrugs in responsive to tumor‐specific endogenous signals, including lower pH, higher reductive conditions, and overexpression of MMP‐2/9. More importantly, the authors demonstrated that the BLPNs were actually effective for cancer immunotherapy, enhancing antitumor immunogenicity and IDO‐1 inhibition to improve the proliferation of CTLs (Figure 19d).

### 3.2 Prodrug Nanomedicine Integrating Chemotherapy and Immune Inhibitor

Some prodrug nanocarriers have shown low antitumor effects in clinical trials, potentially due to slow release of the anticancer agents in the tumor region.^[^ ^43^ ^]^ Although many researchers have adopted strategies to introduce physiologically sensitive bonds, the above‐mentioned dilemmas cannot be well solved due to the heterogeneous of the tumors.^[^ ^159^ ^]^ Compared to normal cells, some tumor cells exhibit higher oxidative stress, but other cancer cells are in reductive stress. This heterogeneity may exist in various types of tumors of different stages as well as different regions within the same tumor.^[^ ^160^ ^]^ Fortunately, redox dual‐responsive linkers, such as disulfide bonds and single thioether bonds, have achieved excellent preclinical outcomes despite tumor heterogeneity.^[^ ^43^, ^161^ ^]^ As previously mentioned, chemotherapy generally causes tumor immunosuppression, further resulting in tumor recurrence or migration.^[^ ^162^ ^]^

Cyclooxygenase‐2 (COX‐2) is an enzyme regulating the synthesis of tumor cell‐derived prostaglandins (PGs), which also serves as a negative immune regulator suppressing the antitumor immunity.^[^ ^163^ ^]^ COX‐2 is overexpressed in a variety of cancer cells for promoting angiogenesis and tumor proliferation. COX‐2 inhibitors may reverse the immunosuppressive tumor microenvironment and serve as an antiangiogenetic therapeutic to inhibit tumor metastasis and reoccurrence.^[^ ^164^ ^]^ To this end, Sharma et al. constructed a SN‐38‐conjugate indomethacin prodrug via a redox‐responsive linker (namely K1). Upon exposure to intracellular GSH and H_2_O_2_, the K1 prodrug was activated inside the tumor cells to release SN‐38 and indomethacin.^[^ ^165^ ^]^ SN38 suppressed tumor growth by inducing apoptosis of the tumor cells, while indomethacin relieving the immunosuppressive tumor microenvironment by suppressing intratumoral secretion of proinflammatory cytokines TNF‐*α* and IL‐6, and enhancing the production of IL‐10 (anti‐inflammatory cytokine).^[^ ^165^ ^]^

**Figure 20:**
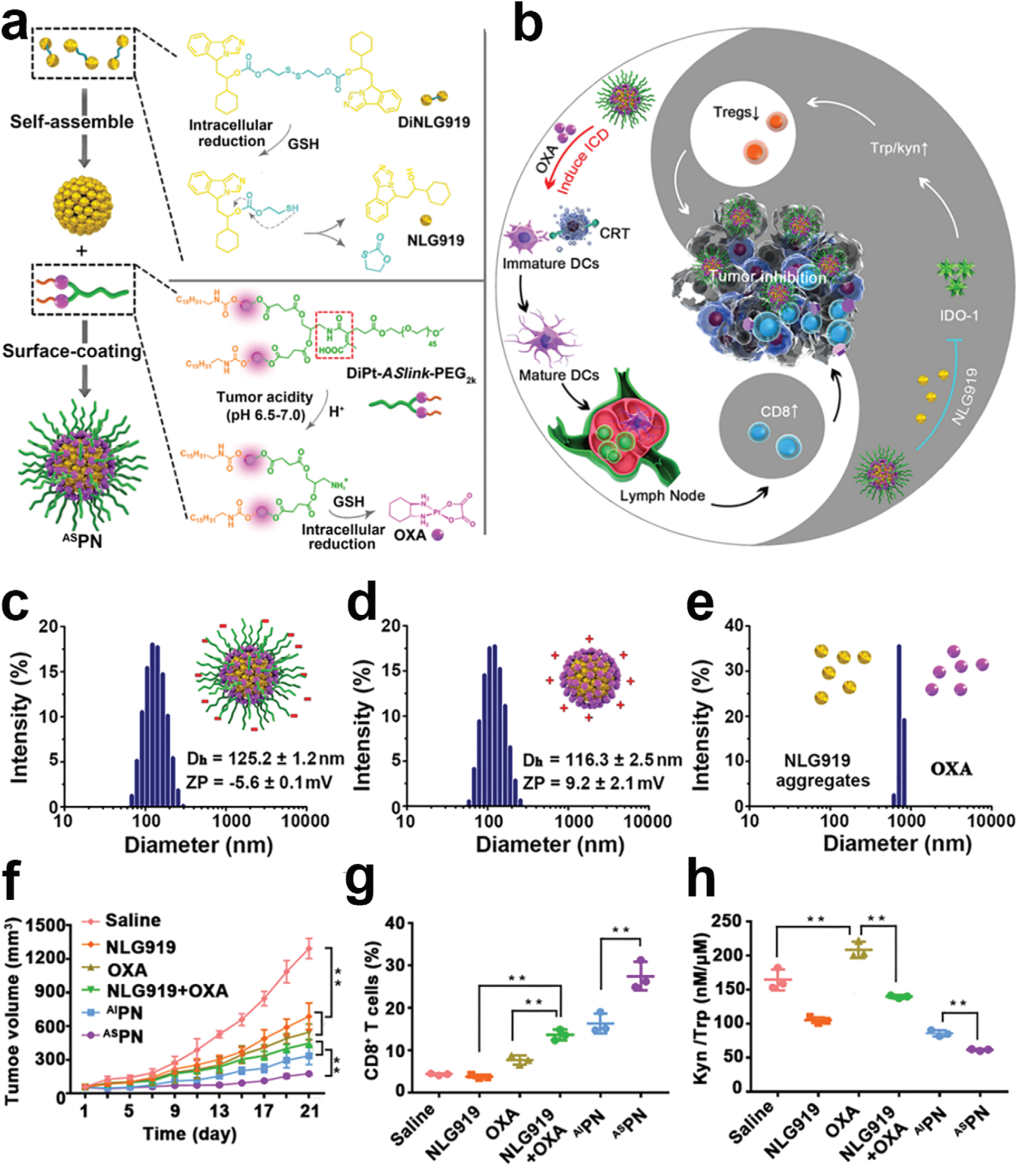
a) Schematic illustration of preparation BCPN. b) Mechanism of BCPN to elicit chemoimmunotherapy. The size change of ^AS^PN in c) pH 7.4 or d) pH 6.5. e) The size of ^AS^PN in pH 6.5 plus GSH. f) Tumor growth curve of tumor‐bearing mice with different formulation. g) Intratumoral infiltration of CD8^+^ T lymphocytes. h) Ratio of Kyn to Trp receiving different treatment. Reproduced with permission.^[^ ^23^ ^]^ Copyright 2018, John Wiley & Sons.

In most cases, hydrophobic small molecule drugs cannot self‐assemble into nanoparticles without the addition of amphiphilic materials. However, Wang et al. discovered that insertion of a disulfide bond into the hydrophobic chemotherapeutic drugs could allow small molecule drugs to self‐assemble into nanoplatforms without other excipients, which greatly improves drug loading and reduces side effects due to excipients.^[^ ^44^, ^166^ ^]^ For example, Feng et al. constructed a NLG919 dimer via a reduction‐responsive disulfide linker, which could simply self‐assemble into a nanoplatform (DiNLG919 NPs) with high drug loading.^[^ ^23^ ^]^ In order to prolong the blood circulation, an acid‐sensitive polyethylene glycol (PEG)‐grafted OXA prodrug was coated on the surface of the DiNLG919 NPs, forming a binary cooperative prodrug nanoparticle (BCPN). When the BCPN reach the tumor site, they are gradually activated by the low pH and the reductive microenvironment to release NLG919 and OXA (**Figure** **20**a,b). Further, the authors also demonstrated that the BCPN had an excellent ability to distinguish different pH and GSH values by detecting the particle size via DLS (Figure 20c–e). Compared to NLG919 or OXA alone, BCPN showed increasing tumor immunogenicity due to OXA‐mediated killing of tumor cells. BCPN significantly reduced the immunosuppressive environment through NLG919 (an IDO inhibitor), which enhanced CTL infiltration and suppressed the Tregs (Figure 20f–h).^[^ ^46^, ^167^ ^]^

**Figure 21:**
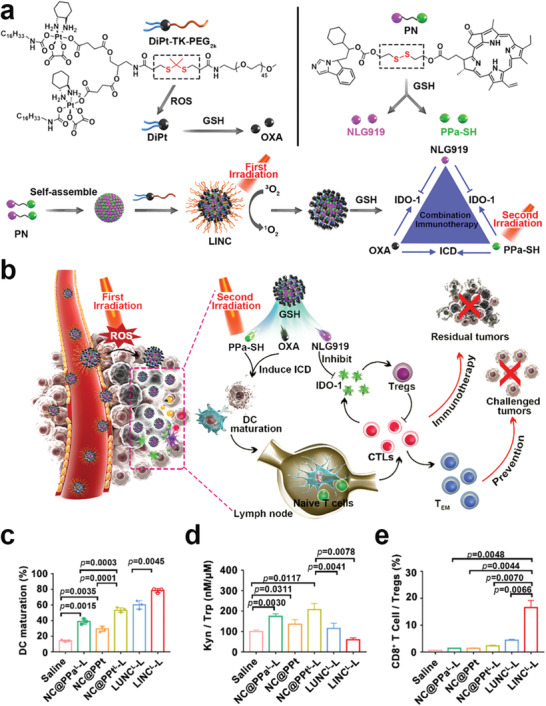
a) Mechanism of LINC for combination immunotherapy. b) Procedure of LINC for chemoimmunotherapy via initiating ICD and reversing immunosuppression. Detection of c) DC maturation, d) Kyn to Trp ratio, e) the infiltration of CD8^+^ with different treatment by flow cytometry. Reproduced with permission.^[^ ^168^ ^]^ Copyright 2019, John Wiley & Sons.

Unlike monotherapy, combination therapy with both PDT and chemotherapy could significantly improve immune responses and better address the problems inherent to tumor heterogeneity. However, PDT and chemotherapy may promote the proliferation of Tregs cells, leading to a strong immunosuppressive condition. Hence, multielement synergistic treatment will greatly improve the effects of treatment, while avoiding the deficiencies that come with monotherapy. To implement this strategy, Feng et al. constructed a tri‐functional prodrug nanoplatform (LINC) that can simultaneously achieve PDT and chemoimmunotherapy.^[^ ^168^ ^]^ The inner core was a reduction‐sensitive heterodimer composed of a photosensitizer (PPa) and an IDO‐1 inhibitor (NLG919),^[^ ^169^ ^]^ and the outer shell was a light‐triggered PEGylated prodrug of OXA (DiPt‐TK‐PEG_2K_). The versatile nanoplatform displayed good serum stability and tumor accumulation in vivo, owing to the protection of the PEGylated shell (**Figure** **21**a). Even after the first wave of laser irradiation, LINC was retained deep in the tumor, and the resulting ROS‐triggered the cleavage of polyethylene glycol, switching particle charge from negative to positive. After the second light treatment, LINC induced extreme ICD due to the rapid release of PPa and OXA (Figure 21b).^[^ ^170^ ^]^ Meanwhile, the released NLG919 relieved tumor immunosuppression caused by up‐regulation of IFN‐*γ*, resulting in increased tumor infiltration of CTLs and inhibiting tumor recurrence (Figure 21c–e). This synergistic self‐amplifying reaction remarkably enhanced drug delivery to deep tumor, meanwhile promoting immunity and improving the antitumor response.

### 3.3 Binary Prodrug Nanomedicine Integrating Gene Therapy with Immunotherapy

Several recent studies had demonstrated that the PD‐L1 immune checkpoint can be silenced by small interfering RNA (siRNA), thereby blocking PD‐1/PD‐L1 pathway and attenuating immune tolerance. However, siRNA is susceptible to serum degradation during blood circulation. Cationic polymer‐based nanovectors were thus employed for systemic siRNA delivery and protecting siRNA from degradation by forming nanocomplexes with siRNA through electrostatic interaction. For instance, Saw et al. reported a polymeric prodrug‐based hybrid nanoplatform for multistage delivery of siRNA‐PD‐L1.^[^ ^37^ ^]^ After reaching the tumor tissues, the multifunctional hybrid nanoplatform rapidly disassociated for fast release of the prodrug‐siRNA complexes. The released complexes were subsequently transported into cytoplasm of the tumor cells, where mitoxantrone was restored by esterase for complex dissociation and siRNA release in the cytoplasm.^[^ ^37^ ^]^

Furthermore, the combination of RNAi‐based PD‐L1 inhibition and some strategies (e.g., photoimmunotherapy) could induce synergistical cancer immunotherapy. For instance, Wang et al. designed an siRNA‐PD‐L1‐loaded acid‐activatable POP micelleplexes by integrating a PS (e.g., PPa)‐conjugated polymer PDPA, amphiphilic cationic polymer OEI‐C14 and siRNA‐PD‐L1 into one single nanoplatform. The POP/siRNA micelleplexes displayed satisfying antitumor performance by eliciting antitumor immune response via PDT and relieving the ITM via siRNA‐based PD‐L1 inhibition.^[^ ^171^ ^]^

## 4 Summary and Perspective

Compared with the conventional therapeutic modalities including chemotherapy, surgical resection, and radiotherapy for cancer treatment, immunotherapy works by activating immune cells and producing memory T cells to regress tumor growth, and immunotherapy has obvious advantages in inhibiting tumor reoccurrence and metastasis.^[^ ^172^ ^]^ However, there are several shortcomings of current cancer immunotherapy, including low immune responses, poor tumor targeting, and severe side effects of the immunotherapeutics.^[^ ^173^ ^]^ Prodrug nanomedicines integrate the advantages of prodrugs and nanotechnology to enhance the efficiency and safety of tumor immunotherapy relative to the conventional nanomedicine.^[^ ^174^ ^]^ Prodrug nanomedicines possess a broad toolkit to improve prodrug stability during circulation and to enhance the bioavailability of codelivered drugs with different pharmacokinetics profiles.^[^ ^175^ ^]^ Upon arriving at the tumor site, prodrug nanomedicines can achieve spatiotemporally controllable restoration of the immunotherapeutics through careful engineering of the stimuli‐responsive linkers that are activated in the tumor microenvironment, thereby more accurately activating immune responses or relieving immunosuppression.

Although the prodrug nanomedicine strategy is generally promising, there are several key considerations for its translation for clinical immunotherapy. First of all, increased research efforts of nanoformulations of prodrugs need to be devoted to address the current shortcomings and broaden the advantages in tumor immunotherapy, and the prodrug nanomedicines should be able to perform precise cancer immunotherapy through rational formulation design. Currently, there are relatively few reports of small molecule prodrugs that self‐assemble into nanostructures that can regulate immunity, compared to a wide variety of publications related to polymeric drug‐conjugated nanomedicines. However, the former has the advantage of utilizing ingredients with well‐defined chemical structures instead of potentially toxic excipients, so self‐assembling small molecule nanomedicines are more likely to yield successful and rapid clinical translation. Therefore, screening and optimization of new prodrug compositions will accelerate the clinical translation of the prodrug‐based nanomedicine. Second, prodrug nanomedicines are more effective in the regulation of immune cells or immune organs, but there is a need for more research on tumor microenvironment and metabolic pathways.^[^ ^176^ ^]^ For instance, novel prodrug nanoplatforms should pay aim to improve cancer immunotherapy by regulating the tumor microenvironment and metabolism. To address this challenge, prodrug nanomedicines can be integrated with a combination of therapeutic regimens for targeting multiple singling pathways. Third, it is highly desirable to develop prodrug nanomedicines with both imaging function and stimuli‐activatable property. Increased efforts should be devoted to developing tumor microenvironment‐activatable chemical bonds. The integration of diagnosis and treatment of tumor immunotherapy is a promising topic for future development of prodrug nanomedicine. For instance, the NIR‐II infrared dyes showed better photostability and tissue penetration ability, they have received increasing attention in the field of diagnosis. Prodrug nanomedicines made by modifying the NIR‐II infrared molecule will further improve the accuracy of tumor immunotherapy.^[^ ^177^ ^]^ Fourth, multidisciplinary research efforts on the topic of immunotherapy is likely the most efficient path forward. The collaborative integration of expertise in immunology, biomedicine, pharmacology, and other fields may help identify new immune targets and pathways and innovative drug delivery systems to achieve high efficiency and low toxicity. Finally, the clinical translation of prodrug nanomedicine will also benefit from the effective communication between the academia, industry, and drug administration of government. Green and cost‐effective chemistry is essential for the scale up of the prodrug nanoparticles. Furthermore, in vitro and preclinical studies under the guideline of drug administration will also be helpful for generating reliable data set for clinical translation of prodrug nanomedicine for cancer immunotherapy.

## Conflict of Interest

The authors declare no conflict of interest.
